# Global functional genomics reveals GRK5 as a cystic fibrosis therapeutic target synergistic with current modulators

**DOI:** 10.1016/j.isci.2025.111942

**Published:** 2025-02-01

**Authors:** Hugo M. Botelho, Miquéias Lopes-Pacheco, Madalena C. Pinto, Violeta Railean, Ines Pankonien, Mariana F. Caleiro, Luka A. Clarke, Vasco Cachatra, Beate Neumann, Christian Tischer, Cristina Moiteiro, Jiraporn Ousingsawat, Karl Kunzelmann, Rainer Pepperkok, Margarida D. Amaral

**Affiliations:** 1 BioISI – Biosystems & Integrative Sciences Institute, Faculty of Sciences, University of Lisboa, Campo Grande, 1749-016 Lisboa, Portugal; 2 Department of Physiology, University of Regensburg, Universitätsstrasse 31, 93053 Regensburg, Germany; 3 Centro de Química Estrutural, Institute of Molecular Sciences, Department of Chemistry and Biochemistry, Faculty of Sciences, University of Lisboa, Campo Grande, 1749-016 Lisboa, Portugal; 4 Cell Biology and Biophysics Unit and Advanced Light Microscopy Facility, European Molecular Biology Laboratory (EMBL), Meyerhofstraße 1, 69117 Heidelberg, Germany; 5 Centre for Bioimage Analysis, European Molecular Biology Laboratory (EMBL), Meyerhofstraße 1, 69117 Heidelberg, Germany

**Keywords:** Cell biology, Functional genomics

## Abstract

Cystic fibrosis (CF) is a life-shortening disease affecting >160,000 individuals worldwide predominantly with respiratory symptoms. About 80% of individuals with CF have the p.Phe508del variant that causes the CF transmembrane conductance regulator (CFTR) protein to misfold and be targeted for premature degradation by the endoplasmic reticulum (ER) quality control (ERQC), thus preventing its plasma membrane (PM) traffic. Despite the recent approval of a “highly effective” drug rescuing p.Phe508del-CFTR, maximal lung function improvement is ∼14%. To identify global modulators of p.Phe508del traffic, we performed a high-content small interfering RNA (siRNA) microscopy-based screen of >9,000 genes and monitored p.Phe508del-CFTR PM rescue in human airway cells. This primary screen identified 227 p.Phe508del-CFTR traffic regulators, of which 35 could be validated by additional siRNAs. Subsequent mechanistic studies established GRK5 as a robust regulator whose inhibition rescues p.Phe508del-CFTR PM traffic and function in primary and immortalized cells, thus emerging as a novel potential drug target for CF.

## Highlights

• Large-scale high-content screen identifies p.Phe508del-CFTR traffic regulator genes
• Validation of hit genes and classification according to ERQC checkpoints
• Chemical inhibition of GRK5 rescues p.Phe508del-CFTR traffic and function
• GRK5 inhibition is additive to CFTR modulators, a potential therapeutic option

## Graphical abstract

**Figure:**
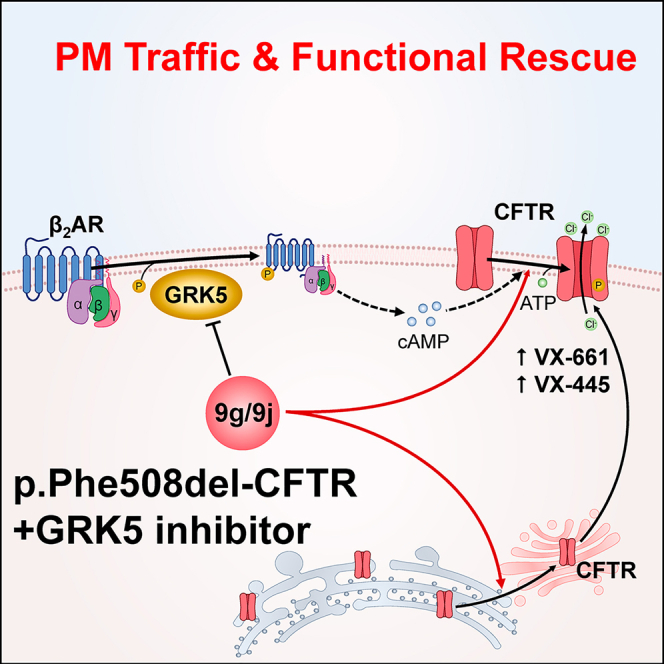

## Introduction

Mutations in the gene encoding the cystic fibrosis transmembrane conductance regulator (CFTR) protein^1^ cause cystic fibrosis (CF), the most common life-shortening autosomal recessive disease (median age at death ∼37 years^2^) affecting >160,000 individuals worldwide.^3^ CFTR is a chloride (Cl^−^) and bicarbonate (HCO_3_^−^) channel expressed at the apical membrane of several epithelial tissues, notably the airways, which constitute the most affected organ in CF. In the airways, CFTR maintains epithelial ion homeostasis, fluid secretion, and hydration of the airway surface liquid (ASL). Dysfunctional CFTR leads to ASL dehydration and persistence of thick mucus, which obstructs the airway and prevents mucociliary clearance. This potentiates chronic inflammation and recurrent bacterial infections that lead to progressive deterioration of lung function, the most frequent cause of morbidity and mortality in CF.

Among the >2,100 CFTR variants so far reported,^4^ the most common one is deletion of residue phenylalanine 508—p.Phe508del (legacy name: F508del-CFTR)—which occurs in ∼80% of CF cases, thus being a preferential target for research and drug development. p.Phe508del-CFTR presents several defects, namely (1) protein misfolding, which is resistant to physiological rescue by endogenous molecular chaperones; (2) deficient PM traffic due to retention of misfolded p.Phe508del-CFTR at several checkpoints of the ER quality control (ERQC) machinery,^5^ which target it for premature degradation via the ubiquitin-proteasome pathway (UPP); (3) impaired function, as the residual amount of p.Phe508del-CFTR that reaches the PM (only in some individuals) has very low activity due to a channel gating defect^6^; and (4) PM instability of rescued p.Phe508del-CFTR, as it is quickly endocytosed and degraded.^7^^,^^8^

Early studies have shown that p.Phe508del-CFTR can be released from the ER to the PM, namely by low temperature expression.^9^ Nevertheless, only recently a highly effective triple combination drug comprising two correctors—tezacaftor (VX-661) and elexacaftor (VX-445), to rescue p.Phe508del-CFTR PM traffic—with a potentiator—ivacaftor (VX-770), to rescue the gating defect—was shown to be efficacious in CF individuals bearing one or two p.Phe508del alleles.^10^^,^^11^ Even so, the maximum clinical benefit of this highly effective drug is estimated at an average ∼14% lung function improvement (ppFEV_1_), with some individuals with less than 5% ppFEV_1_ increase.

Evidence supports the binding of tezacaftor and the first-generation corrector lumacaftor (VX-809) to CFTR^8^^,^^12^^,^^13^ where they restore the interaction between the first nucleotide binding domain (NBD1) and the fourth intracellular loop (ICL4).^14^ However, the mechanism of action (MoA) for the elexacaftor corrector remains elusive. Modeling studies have suggested binding to the same lumacaftor/tezacaftor site and/or to a distinct site at the first membrane spanning domain (MSD1).^15^ The identification of the machinery assessing CFTR folding at ERQC checkpoints^16^^,^^17^ will likely shed light on the MoA of this and future traffic-rescuing drugs.^18^ Likewise, global knowledge on ERQC intervenients is expected to contribute toward the rational development of improved therapeutic strategies aimed at rescuing p.Phe508del-CFTR, as well as other CFTR folding variants, and/or other misfolded proteins causing traffic disorders.^19^

The complex ERQC process by which CFTR folding is assessed has been proposed to consist of at least four sequential checkpoints,^16^^,^^20^^,^^21^ namely through (1) interaction with the Hsp70 chaperone machinery where most p.Phe508del-CFTR is degraded; (2) the calnexin folding cycle; (3) exposure to an unidentified machinery retaining arginine-framed tripeptides (AFT: Arg-X-Arg), four of which are present in CFTR; and (4) interaction of the CFTR di-acidic exit code (Asp^565^-Ala-Asp^567^) with Sec23/Sec24 for ER exit through coat protein (COP)-II-coated vesicles.^22^^,^^23^ Failure to overcome one or more checkpoints inevitably leads to CFTR retention in the ER and subsequent degradation via UPP. Most of the knowledge on the ERQC checkpoints was obtained through studies with second-site "revertant" variants that render p.Phe508del-CFTR less susceptible to UPP at selected checkpoints.^17^^,^^24^^,^^25^ Revertants also enabled dissection of the MoA of corrector drugs.^14^ The factors that retain p.Phe508del-CFTR at each ERQC checkpoint have been elucidated by hypothesis-driven approaches, being thus likely that many remain unidentified.

Here, we hypothesized the existence of still unidentified cellular factors retaining p.Phe508del-CFTR in the ER that might become potential drug targets for CF as well as for other diseases caused by misfolded proteins. To identify these factors on a global scale, we applied a cell-based high-throughput (HT) microscopy approach using loss-of-function assays to screen a druggable genome library comprising 27,310 small interfering RNAs (siRNAs) for PM rescue of p.Phe508del-CFTR.^26^^,^^27^ After carrying out hit validation, mechanistic classification according to ERQC checkpoint targets, and assessment of CFTR rescue additivity with state-of-the-art folding corrector drugs, we eventually selected five kinase proteins and studied their MoA. Of these, G-protein-coupled receptor kinase 5 (GRK5) was selected as the hit with highest druggable potential, based on the rescue of p.Phe508del-CFTR traffic and function upon its inhibition by either siRNAs or recently reported selective inhibitors—9g or 9j—in a manner additive to CFTR correctors. We thus propose GRK5 as a potential therapeutic target for CF and discuss the mechanism by which GRK5 inhibition may rescue p.Phe508del-CFTR from its ER retention.

## Results

### Primary screen to identify global regulators of p.Phe508del-CFTR traffic

**Figure 1:**
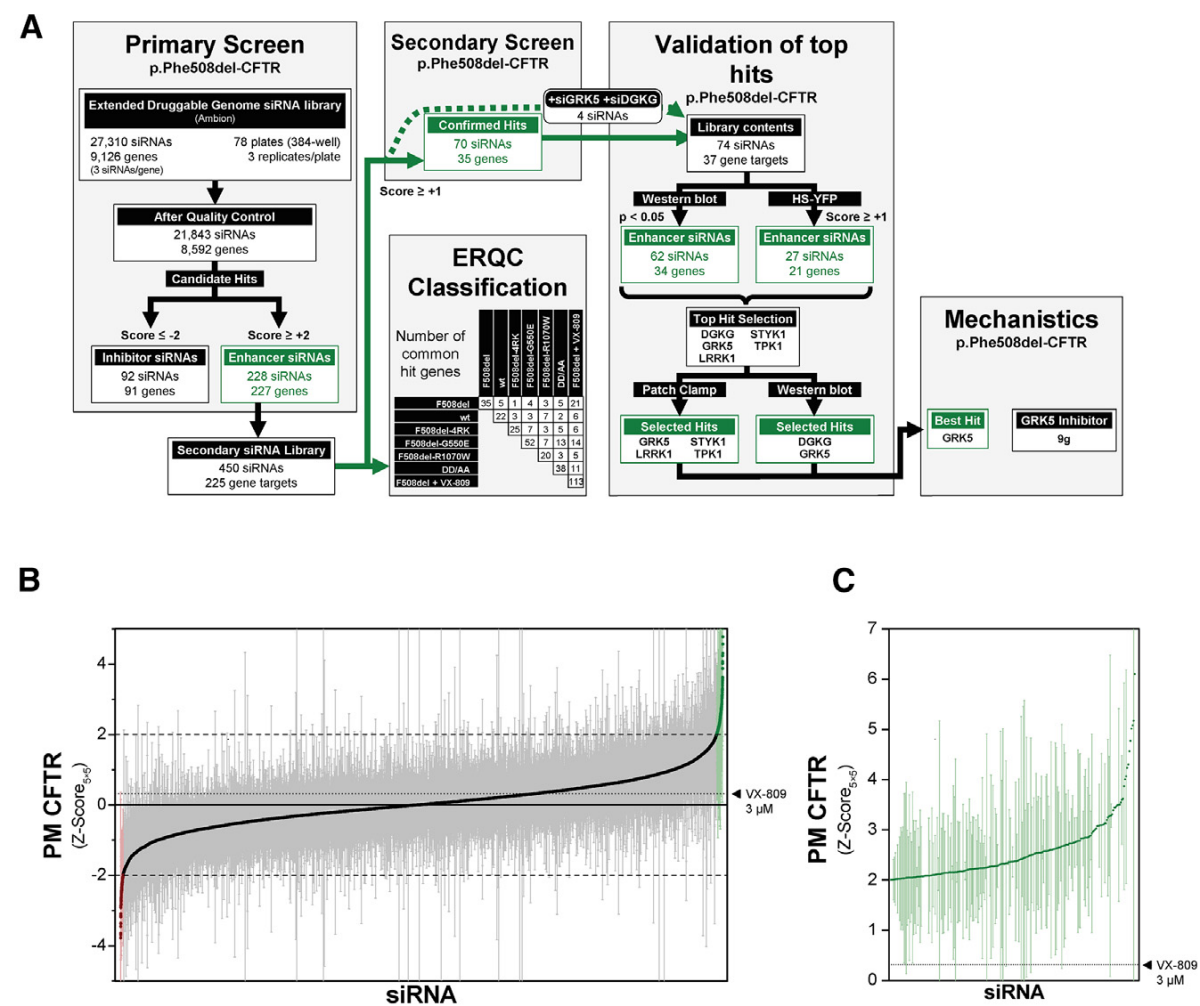
Workflow of siRNA-based p.Phe508del-CFTR traffic screens (A) The primary HCS was based on screening the Ambion extended druggable genome siRNA library (9,126 genes, each targeted by three individual siRNAs) with a CFBE cell line expressing the mCherry-Flag-p.Phe508del-CFTR traffic reporter. An siRNA subset was discarded due to off-target effects or failed assay plate preparation. The primary screen highlighted 228 hit siRNAs—targeting 227 genes—which produced significant increases in p.Phe508del-CFTR PM delivery. Most primary hits (225 genes) were re-screened with additional siRNAs (2 siRNAs/gene). A total of 35 gene hits, targeted by 70 siRNAs, were confirmed. For validation, an siRNA library targeting the 35 confirmed hit genes as well two additional kinases (DGKG, GRK5) that were among the primary hits was assembled. Significant increases in p.Phe508del-CFTR glycosylation (western blot) or function (HS-YFP) were achieved by knocking down, respectively, 34 or 21 of the 37 genes. The overlap between YFP and WB is 20 genes and 25 siRNAs. Downstream studies focused on five kinases, selected for their druggability. GRK5 was selected as the best hit, and additional studies with a specific small molecule inhibitor were performed. In parallel, reporter cell lines expressing wt-CFTR, the DD/AA-CFTR variant, or genetic p.Phe508del-CFTR revertants affecting ERQC checkpoints were used to screen the confirmation siRNA library, thereby classifying hit genes according to their dependence on different ERQC mechanisms. For simplicity, the summary matrix uses single letter amino acid codes. (B) Waterfall plot with *Z* score_5×5_ for PM p.Phe508del-CFTR (median ± SEM) obtained with all siRNAs analyzed in the primary screen and (C) inset showing the subset of 228 primary siRNAs that significantly increase PM p.Phe508del-CFTR. Dotted lines show the positive (*Z* score_5×5_ > +1), negative hit threshold (*Z* score_5×5_ < −1), or the readout for cells treated with 3 μM VX-809. For most siRNAs *n* = 3 biological replicates (see Data S1).

To identify genes retaining p.Phe508del-CFTR in the ER, we deployed an established high-content screening (HCS) pipeline based on a Tet-inducible p.Phe508del-CFTR traffic reporter (mCherry-Flag-p.Phe508del-CFTR) stably expressed in human airway cells.^26^ This pipeline was used to screen the Ambion Human Extended Druggable Genome siRNA library (27,310 siRNAs targeting 9,126 genes; Figure 1A; Data S1A). Our main readout was the amount of p.Phe508del-CFTR at the PM, since KD of p.Phe508del-CFTR ER retention factors are expected to increase the amount of this variant at the cell surface and, consequently, channel activity. Data were expressed as a *Z* score that took the 5 × 5 neighboring wells as baseline (*Z* score_5×5_; Figure S1). After filtering out siRNAs with multiple or non-protein coding targets, plates with unsuccessful siRNA coating and non-representative cells, 21,843 siRNAs targeting 8,592 genes were scored regarding p.Phe508del-CFTR PM levels. Targeted genes were classified as positive or negative hits, whenever their siRNA KD resulted in significant enhancement (*Z* score_5×5_ ≥ +2) or reduction/inhibition (*Z* score_5×5_ ≤ −2) of PM p.Phe508del-CFTR, respectively. Positive gene hits are the putative p.Phe508del-CFTR retention factors of interest. Using this stringent criterion, we identified 227 genes, targeted by 228 siRNAs, as positive hits (Figures 1B and 1C; Data S1B). Only one gene had two distinct siRNAs leading to p.Phe508del-CFTR PM rescue, UQCRFS1 (ubiquinol-cytochrome c reductase, Rieske iron-sulphur polypeptide 1). Conversely, we could still identify 91 genes, targeted by 92 siRNAs, as negative hits (Data S1C). In the primary screen, p.Phe508del-CFTR PM levels were highly correlated (Spearman’s ρ = 0.93, *p* < 2.2x10^−16^) with CFTR traffic efficiency (the Flag/mCherry fluorescence ratio), which was also computed.

To gain insight into the nature of primary hits, we performed overrepresentation tests on the Gene Ontology (GO) terms associated with these genes, including positive (Figures S2A–S2C; Data S2A–S2C) and negative hits (Figures S2D–S2F; Data S2C–S2F). In the positive hit genes, we found overrepresentation of the terms PM (cell component), kinase activity, ionic binding and transport, and adenylyl nucleotide binding (molecular function), and phosphorylation (biological process). These terms suggested that several positive hits are other PM proteins, possibly competing with CFTR to access the secretory pathway through the ERQC machinery. Regarding negative hits, GO revealed an enrichment in PM proteins (cellular component), enzymes, and metal binding, pointing to biological pathways possibly favoring p.Phe508del-CFTR traffic.

### Secondary screen to confirm primary screen hits as retention factors of p.Phe508del-CFTR traffic

**Figure 2:**
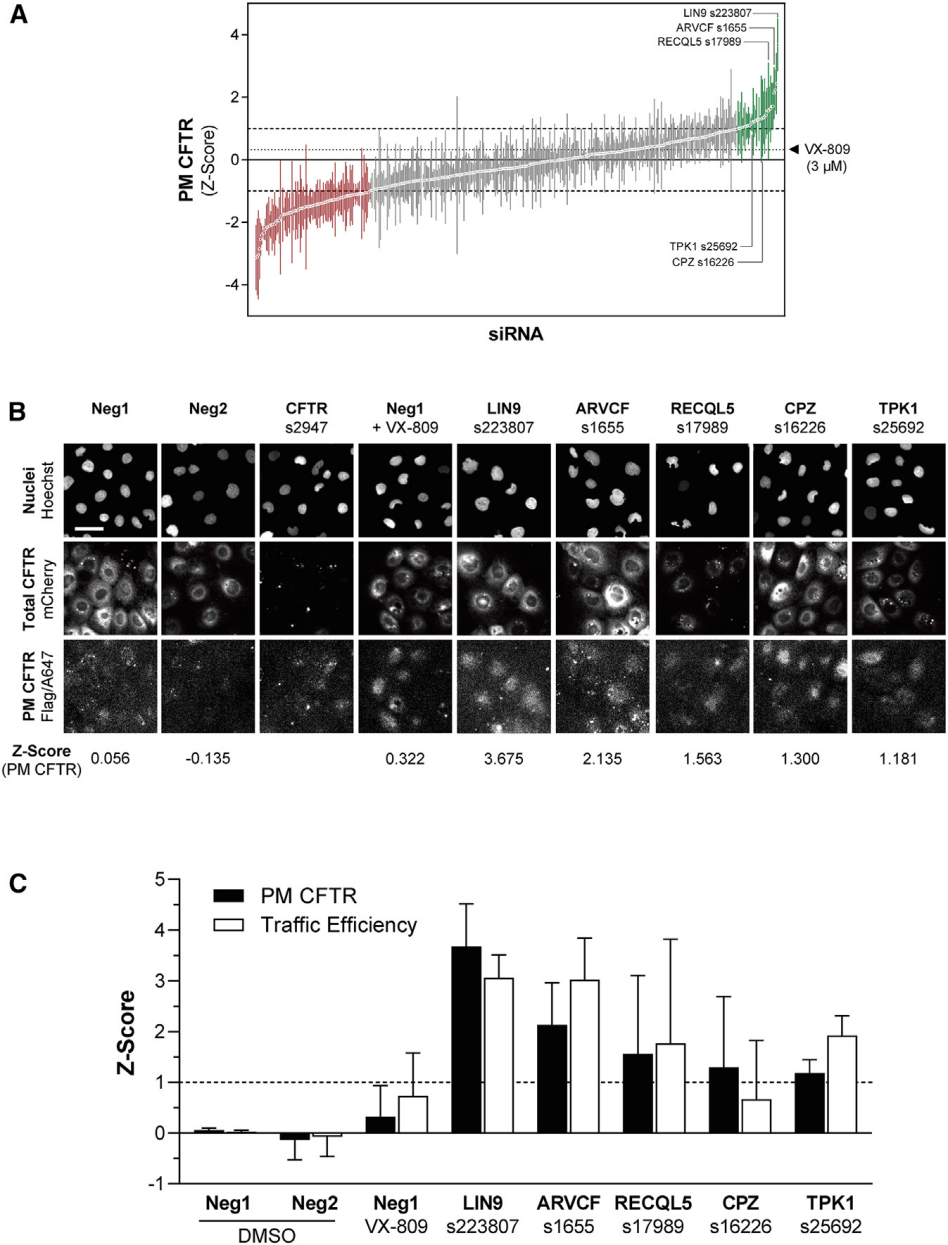
Overview of the confirmation siRNA screen (A) Waterfall plot of p.Phe508del-CFTR PM localization *Z* scores. Values are median ± SEM. Negative (*Z* score <1). Negative and positive hits are shown in red or green, respectively. Selected positive hits from (B) are shown. (B) Representative microscopy images of controls and selected hit genes. Neg1 is the non-targeting siRNA used as negative control for *Z* score calculation. Neg2 is another non-targeting siRNA that yields CFTR PM *Z* score close to the baseline. VX-809 (3 μM) was added to selected wells containing Neg1 as a positive control. The loss of CFTR expression (mCherry) in CFTR siRNA-treated cells reports on a high transfection efficiency. Gene symbols, the respective siRNAs catalog numbers, and median *Z* scores are shown. Scale bar: 50 μm. (C) *Z* score for the PM and traffic efficiency quantification, shown as median ± SEM. For most siRNAs *n* = 3 biological replicates (see Data S3).

Primary screen hits were rescreened using distinct siRNA molecules. A secondary library of 450 siRNAs targeting 225 of the 227 positive hit genes was assembled (siRNAs were unavailable for two genes), and p.Phe508del-CFTR PM levels were scored by the same traffic assay (Figure 2A). In this assay, we considered negative hits when *Z* score ≤ −1 and positive hits when *Z* score ≥ +1. We could confirm 35 genes, each targeted by 2 distinct siRNAs, whose KD robustly increased p.Phe508del-CFTR PM levels in both the primary and secondary screening rounds (Table S1; Data S3). This stringent criterion equates to a 15% hit validation rate. When inhibited, these “confirmed gene hits” robustly increased p.Phe508del-CFTR PM availability (Figures 2B and 2C), further supporting their role as p.Phe508del-CFTR retention factors. Notably, in both screening rounds the confirmed hits rescued p.Phe508del-CFTR to a larger extent than the clinical drug VX-809 (lumacaftor, 3 μM). Enrichment analysis of these 35 hit genes did not reveal any overrepresentation of GO terms, likely due to the small sample size.

### Validation of p.Phe508del-CFTR traffic regulators by processing and functional assessment

**Figure 3:**
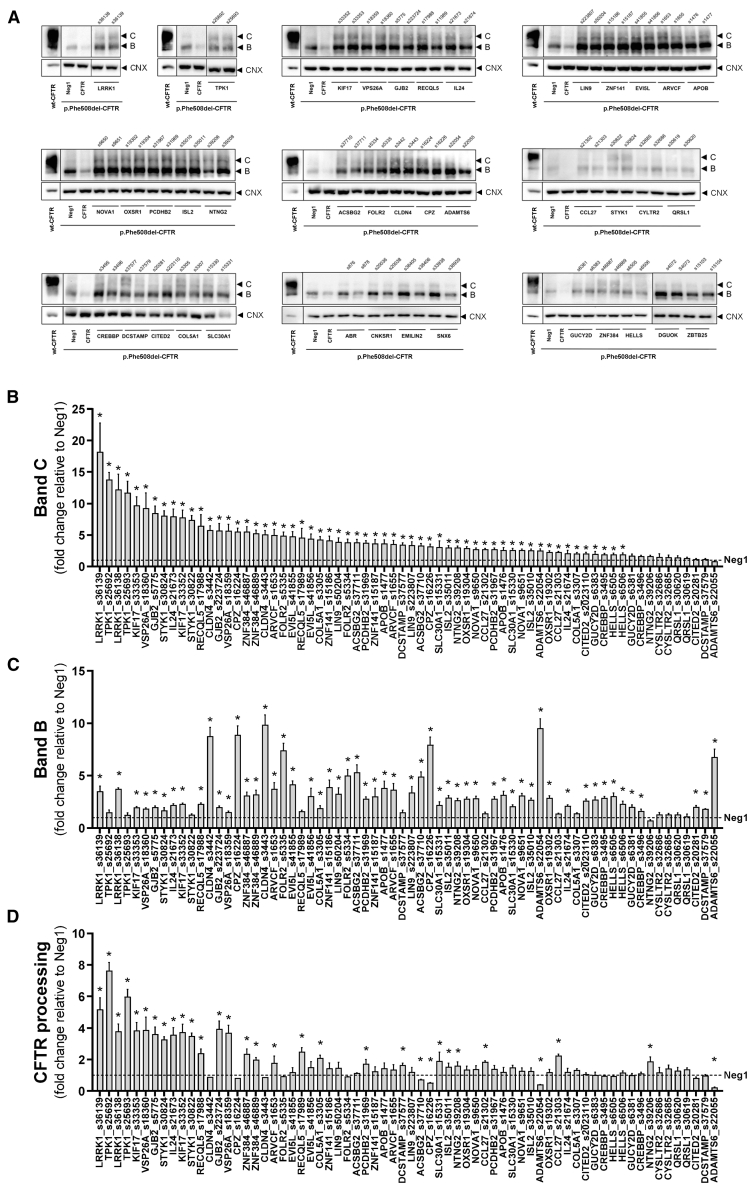
Effect of hit siRNAs on p.Phe508del-CFTR processing assessed by WB (A) CFBE cells overexpressing p.Phe508del-CFTR were transfected with siRNAs targeting 34 of the 35 confirmed hit genes (LDLRAD could not be included) and analyzed by WB for processing to confirm effects on CFTR traffic rescue. Wt-CFTR was included for reference. Densitometric quantification of the fully glycosylated band C (B), immature, core glycosylated band B (C), and CFTR processing [C/(B + C)] (D), normalized to the corresponding loading controls (mean ± SD). “∗” indicates statistical significance from Neg1-treated cells (*p* < 0.05, one-way ANOVA followed by Dunnett’s post-hoc test, *n* = 3 biological replicates). Of the 68 tested siRNAs, 58 showed significant band C rescue and 27 showed significantly increased processing. For 26 siRNAs simultaneous increases in band C and processing were observed. Gel lanes were reordered and juxtaposed for presentation consistency and clarity.

Since the 35 confirmed hits were defined solely by the microscopy-based assay, we performed complementary experiments to further validate them as p.Phe508del-CFTR regulators. CFTR is a glycoprotein that, with an ER-specific core-glycosylated form, is observable in western blotting (WB) as a “band B.” ER exit is followed by processing in the Golgi apparatus to yield a high-molecular-weight fully glycosylated form, “band C.” Through densitometric analysis, the fraction of processed CFTR can be determined as [C/(B + C)]. We transfected CFBE cells expressing untagged p.Phe508del-CFTR with siRNAs targeting each of the 35 confirmed hits, performed WB and investigated KDs that increased band C over the control (Figure 3; Table S2). These experiments validated 32 of the 35 genes (91%) as putative regulators of p.Phe508del-CFTR PM traffic (statistically significant band C increases over control for at least one siRNA). Of these, 17 siRNAs also increased processing (ARVCF, CCL27, COL5A1, DCSTAMP, GJB2, IL24, ISL2, KIF17, LRRK1, NTNG2, PCDHB2, RECQL5, SLC30A1, STYK1, TPK1, VSP26A, ZNF384). This remarkable validation rate, together with the above stringent hit selection criterion, prompted us to examine whether the genes targeted by siRNAs that scored just below the hit threshold in the secondary HCS could also significantly rescue p.Phe508del-CFTR processing. Therefore, WB analysis after knocking down 18 additional gene hits recovered from the primary screen, but which were not confirmed in the secondary screen, showed for all a significant band C increase versus the control (Figure S3; Table S2). Interestingly, among the 50 genes validated in the WB assay, five kinases yielded some of the highest band C readouts: LRRK1, TPK1, STYK1, GRK5, and DGKG. The LRRK1, TPK1, and STYK1 KDs produced p.Phe508del-CFTR rescue in both HCS rounds and WB assays. To determine the physiological relevance of all 53 genes analyzed through HCS and/or WB, we assessed their expression in human native lung tissue by RT-PCR as well as in wt-CFTR-expressing CFBE cells (Data S4). All but four genes (CITED2, KIF17, ACSBG2, GUCY2D) were expressed in human native lung tissue (92.4%). Expression of three genes (APOB, ACSBG2, GUCY2D) could not be detected in CFBE cells. Of relevance, the 18 genes confirmed exclusively by WB were expressed in both specimens.

**Figure 4:**
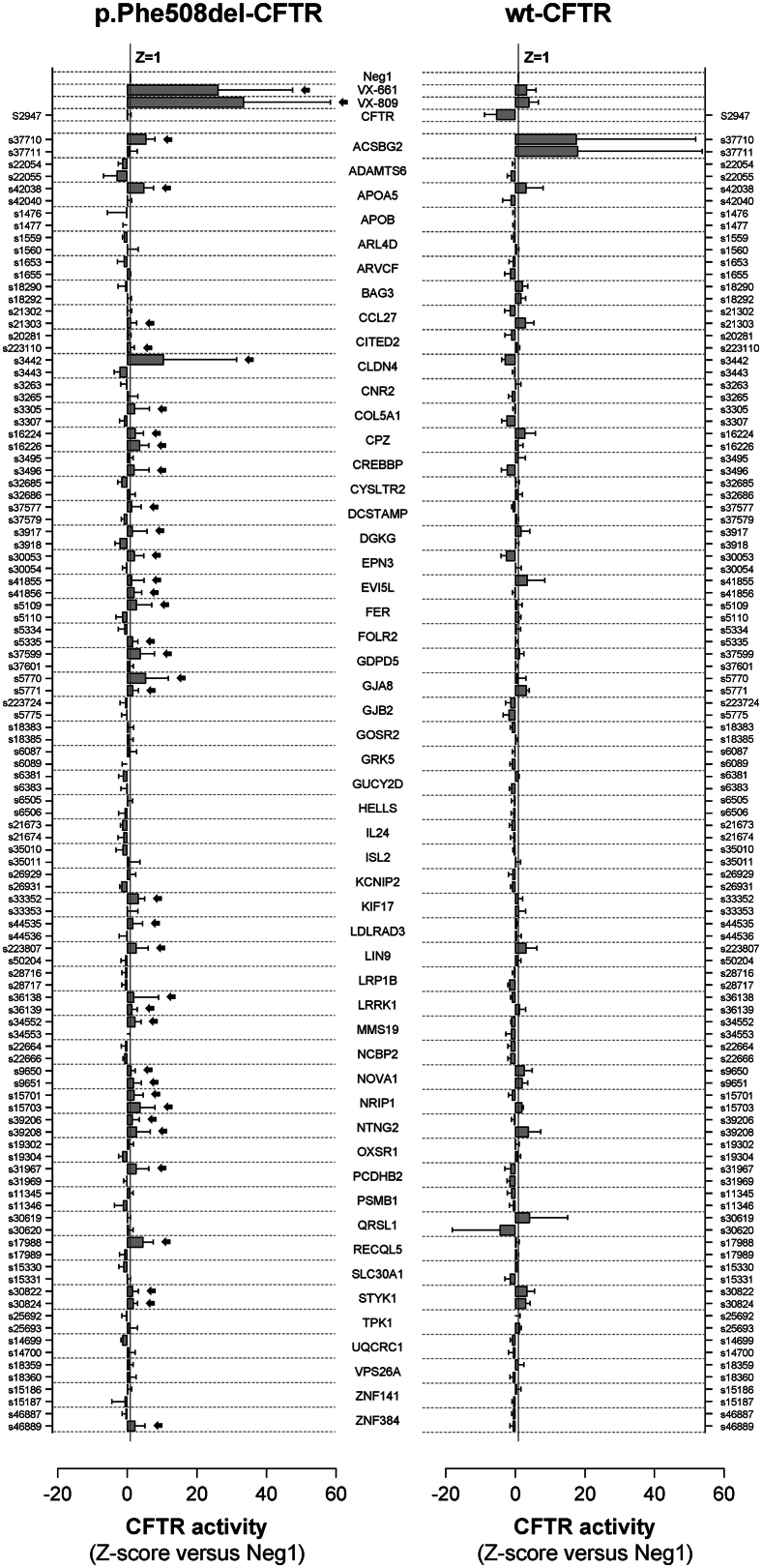
Quantification of CFTR function through the HS-YFP quenching assay CFBE cells expressing p.Phe508del- or wt-CFTR were transfected with siRNAs targeting the 53 confirmed hit genes, and ionic transport was measured via the HS-YFP quenching assay. Gene targets and siRNA IDs are shown. Plot values are quenching rate *Z* score versus the negative control (mean ± SD, n = 2–8 biological replicates). Vertical gray lines mark the Z = 1 threshold applied for hit identification. Hit siRNAs for p.Phe508del-CFTR function rescue are shown with arrows. Numerical data available in Data S5.

To determine whether the 53 confirmed hit genes (considering HCS and WB) also rescue p.Phe508del-CFTR Cl^−^ conductance, we performed the halide-sensitive YFP (HS-YFP) fluorescence quenching assay after siRNA KDs as in the secondary screen (Figure 4, Data S5). Cells expressing wt-CFTR were also tested. The assay validated 20 of the 35 HCS confirmed hit genes targeted by 26 siRNAs, whose KD significantly enhanced p.Phe508del-CFTR function above control (*Z* score > +1), including LRRK1 and STYK1, but not TPK1. Regarding the 18 additional genes, 13 of them were tested, and KD of 7 genes by 9 siRNAs enhanced p.Phe508del-CFTR function. Overall, KD of 12 of these 27 genes enhanced p.Phe508del- but not wt-CFTR function: CITED2, CLDN4, COL5A1, CREBBP, DCSTAMP, EPN3, FER, FOLR2, KIF17, LDLRAD3, PCDHB2, RECQL5, and ZNF384 (Data S5G). Conversely, only two gene KDs enhanced wt- but not p.Phe508del-CFTR function: QRSL1 and TPK1.

### Classification of traffic regulators regarding ERQC checkpoints and CFTR global datasets

We hypothesized that some of the p.Phe508del-CFTR traffic hits could be constituents of the ERQC checkpoints. To test this hypothesis, we assessed the effects of their KD on the traffic of (1) wt-CFTR; (2), p.Phe508del-CFTR revertant variants (p.Phe508del-p.Gly550Glu-CFTR [legacy: F508del-G550E-CFTR], p.Phe508del-p.Arg1070Trp-CFTR [legacy: F508del-R1070W-CFTR], p.Phe508del-4RK-CFTR); or (3) a traffic-inhibiting CFTR variant (DD/AA-CFTR), all stably expressed in CFBE cell lines as double-tagged (mCherry/Flag) Tet-inducible traffic reporters. This “classification screen” aimed at assigning each hit gene to one or more ERQC checkpoints (Figure 1A). These five cell lines were reverse transfected with the secondary library, and a positive effect was considered when at least one siRNA significantly increased CFTR PM levels (*Z* score > +1, Data S6). Treatment with VX-809 was used in parallel as a positive control. When excluding control siRNAs, we obtained a positive effect for 22 genes for wt-CFTR, 52 genes for p.Phe508del-p.Gly550Glu-CFTR, 20 genes for p.Phe508del-p.Arg1070Trp-CFTR, 25 genes for p.Phe508del-4RK-CFTR, and 38 genes for DD/AA-CFTR (Data S6). To ascertain specificity, we searched for gene KDs that enhanced CFTR PM delivery in wt-, p.Phe508del revertants, and DD/AA-CFTR but not in p.Phe508del-CFTR. We identified such an effect in 13 genes for wt-CFTR, 40 genes for p.Phe508del-p.Gly550Glu-CFTR, 14 genes for p.Phe508del-p.Arg1070Trp-CFTR, 22 genes for p.Phe508del-4RK-CFTR, and 32 genes for DD/AA-CFTR (Table S3). Conversely, to ascertain ERQC involvement, we searched for gene KDs, which had opposite effects on PM delivery of p.Phe508del-CFTR (enhancers) and other variants (inhibitors). We identified such an effect on 19 genes for wt-CFTR, 5 genes for p.Phe508del-p.Gly550Glu-CFTR, 14 genes for p.Phe508del-p.Arg1070Trp-CFTR, 9 genes for p.Phe508del-4RK-CFTR, and 4 genes for DD/AA-CFTR (Table S4). This last set of genes represented candidate p.Phe508del-CFTR ER retention factors that act at the ERQC checkpoints modulated by these CFTR variants.

To obtain additional information on the specificity and MoA of the 53 confirmed hit genes, we performed hierarchical clustering of the classification screen data (Figure S4A). We considered all hit siRNAs and identified five clusters, where gene KD produced the following general phenotypes: cluster 1: strong inhibition of wt-, p.Phe508del-p.Arg1070Trp- and p.Phe508del-4RK-CFTR traffic, with enhancement of p.Phe508del-CFTR traffic with or without VX-809; cluster 2: strong enhancement of p.Phe508del- regardless of VX-809, p.Phe508del-p.Gly550Glu-, and DD/AA-CFTR and mixed effects on other CFTR variants; cluster 3: moderate enhancement of all variants; cluster 4: preferential enhancement of p.Phe508del-CFTR regardless of VX-809, with moderate effect on other variants; cluster 5: preferential enhancement of p.Phe508del-CFTR, sometimes maximized by VX-809 or the p.Gly550Glu revertant, with moderate or inhibitory effects on other variants.

This classification suggested that genes in Clusters 1 and 2 are highly specific as p.Phe508del-CFTR traffic regulators, whereas those in the remaining clusters seem less specific, given the overall milder or antagonistic KD phenotype on other CFTR variants. Cluster 3 gathers general CFTR ER retention factors, unrelated to ERQC checkpoints, given the mostly enhanced traffic readout on all variants. The rescue of p.Phe508del-CFTR PM localization by siRNAs targeting Cluster 5 genes seems to be additive with NBD1 folding, given the enhanced rescue when combined with either VX-809 (e.g., siGJB2) or p.Gly550Glu (e.g., siEVI5L, siLRRK1), a revertant proposed to stabilize NBD1 conformation and dimerization. Involvement in ERQC checkpoints is further suggested by the inhibitory effect of most cluster 5 gene KDs in wt-CFTR traffic. Interpretation of the contribution of most genes is not clear, possibly due to genes targeting more than one checkpoint or non-ERQC pathways (Figure S4B). Nevertheless, the kinases in this gene set (LRRK1, TPK1, and STYK1) seem to be specific p.Phe508del-CFTR regulators, given that rescue is not maximized when assessing non-p.Phe508del-CFTR variants, with the notable exception of LRRK1 in p.Phe508del-p.Gly550Glu-CFTR (Figure S4C).

To ascertain the overall pathways targeted by the p.Phe508del-CFTR traffic regulators identified here, we compared the primary and secondary screen hits (including the 53 genes validated by HCS or WB) with published datasets of global CFTR interactors and CF disease modulators (Figure S4D), namely p.Phe508del-CFTR interactome in HBE41o^−^ cells^28^; p.Phe508del-CFTR interactome in CFBE41o^−^ cells^29^^,^^30^; genes whose silencing significantly rescued p.Phe508del-CFTR channel activity^31^; candidate modifier genes of CF lung disease^32^; ENaC activating genes in A549 cells^33^; and protein secretion machinery in HeLa cells.^34^ Partial interactomes (e.g., PM-CFTR only^35^) were not included in this analysis so as to widen the scope as much as possible. We found 221 instances where the same gene was mentioned in pairs of published datasets. When inspecting our primary and secondary screen hits, there were 32 instances where these hits were mentioned in other studies. Among the 53 hits, only 8 appear in other datasets: ARVF and NRIP1 (Dang et al.), COL5A1 and MYH14 (Reilly et al.), and CNR2, FER, GOSR2, and OXSR1 (Simpson et al.). When considering the five kinases mentioned above, only GRK5 was found in another dataset (Almaça et al.), indicating that knocking down GRK5 may not only rescue p.Phe508del-CFTR traffic but also inhibit ENaC activity. The low number of genes common to this study and the dataset selection suggests that our HCS platform is sensitive to identify CFTR regulators that may not directly interact with CFTR or may still be unidentified elements of CFTR regulatory networks.

### Selection of five kinases as top hits of high druggability potential

**Figure 5:**
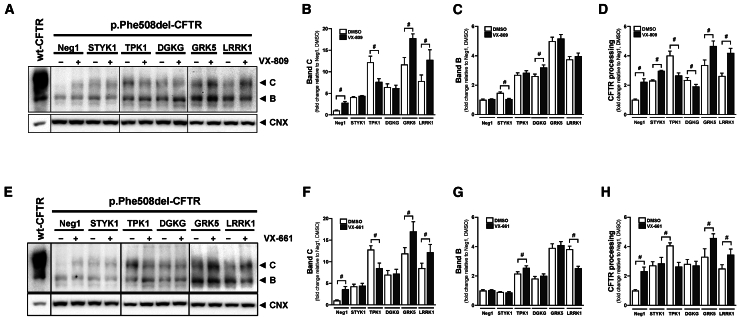
Additivity of hit kinase KD with CFTR correctors on p.Phe508del-CFTR processing rescue CFBE cells expressing p.Phe508del-CFTR were transfected with Neg1 or siRNAs targeting STYK1, TPK1, DGKG, GRK5, or LRRK1 and incubated for 24 h with DMSO (vehicle), 3 μM VX-809 (A–D), or 5 μM VX-661 (E–H). Representative WB membranes (A, E) and densitometric quantification of CFTR and C normalized to the calnexin loading control (B–D and F–H). “#” indicates statistical significance from the corresponding DMSO control (*p* < 0.05 in double-tailed unpaired *t* test, *n* = 3 biological replicates). Plot values are mean ± SD. Gel lanes were reordered and juxtaposed for presentation consistency and clarity.

Since we aimed to identify druggable targets enhancing p.Phe508del-CFTR PM localization, we focused on kinase genes, given their amenability to small-molecule inhibition, which makes them preferential drug targets. Thus, among the hit genes identified, five kinases were selected for further studies: LRRK1, TPK1, STYK1, GRK5, and DGKG (Figure 1A). LRRK1, TPK1, and STYK1 were hits in the primary and secondary HCS, as well on the WB validation. GRK5 and DGKG were hits with high PM scores in the primary screen (4.6 and 3.4, respectively; Data S1B) and with a robust p.Phe508del-CFTR rescue on WB (18- and 12.3-fold change in band C, respectively; Table S2). To determine whether these kinases act through the same MoA as CFTR corrector drugs, we performed KD of each kinase in CFBE cells expressing non-tagged p.Phe508del-CFTR with and without combined administration of VX-809 (3 μM) and VX-661 (5 μM) (Figure 5). Band C rescue was additive with correctors for GRK5 and LRRK1 and unaffected by DGKG or STYK1 KD. TPK1 KD reduced band C levels in the presence of correctors.

**Figure 6:**
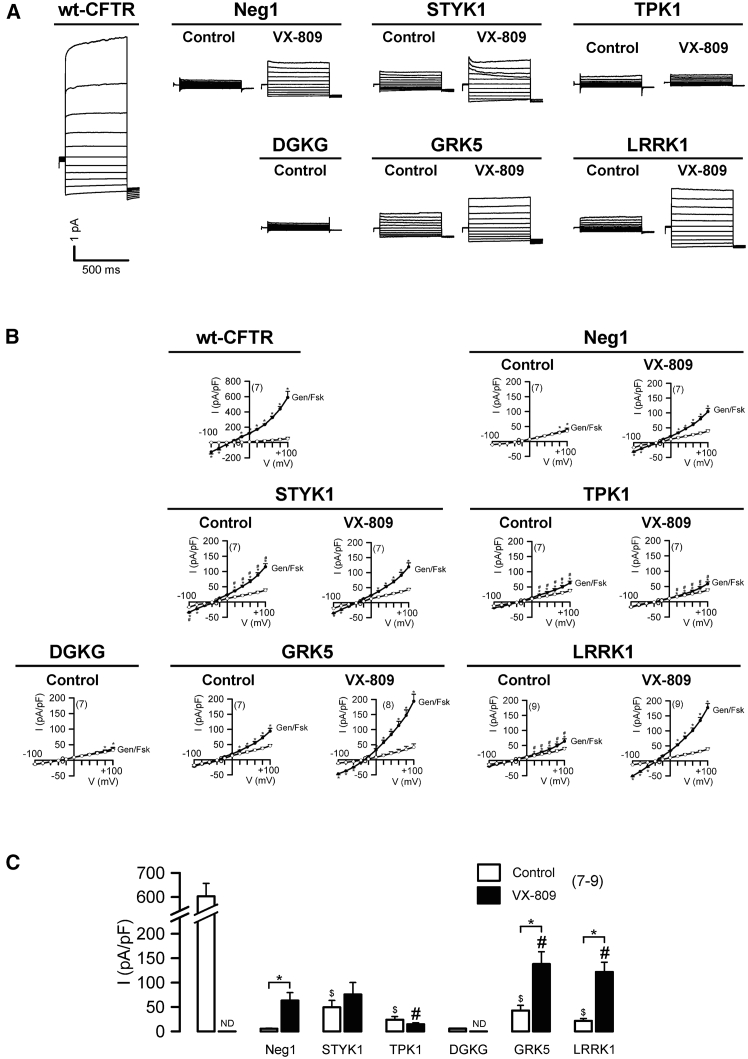
Whole-cell currents of CFBE cells overexpressing p.Phe508del-CFTR and treated with siRNAs targeting hit kinases (A) Whole-cell overlay currents (Vc = ± 100 mV, steps 20 mV) activated by 25 μM Genistein + 2μM Fsk (Gen/Fsk) in CFBE wt-CFTR cells (non-treated) or CFBE p.Phe508del-CFTR cells transfected with Neg1 or siRNAs targeting STYK1, DGKG, TPK1, GRK5, or LRRK1 with and without incubation for 24 h with 3 μM VX-809. (B) Current/voltage (I/V) curves −100 mV to +100 mV for each siRNA in Ringer (white) or after stimulation with 25 μM Genistein + 2μM Fsk (Gen/Fsk) (black). All solutions contained 50 nM TRAM 34, a potassium (K^+^) channel inhibitor to discard K^+^ currents, and the number (n) of experiments is indicated in each graph. (C) Current densities obtained at Vc = +100 mV. Delta of the average of Gen/Fsk-induced current densities. “$” indicates statistical significance of Gen/Fsk-stimulated currents of the respective siRNA vs. Neg1 (*p* < 0.05 in unpaired *t* test), “#” siRNA+VX-809 vs. Neg1+VX-809 (*p* < 0.05 in unpaired *t* test), “∗” siRNA control vs. siRNA + VX-809 (*p* < 0.05 in unpaired *t* test). Values are mean ± SD. Numbers within parenthesis are the replicate count. ND: not detected.

To further examine the pharmacological relevance of the five kinases, we assessed the effects of their down-regulation in whole-cell patch-clamp experiments. Control experiments showed the characteristic outwardly rectifying current of wt-CFTR^36^^,^^37^^,^^38^^,^^39^ (Figure S5). The KD of four kinases (STYK1, TPK1, GRK5, and LRRK1) significantly rescued p.Phe508del-CFTR function, with GRK5 and STYK1 being the most effective and DGKG having no effect (Figure 6). The presence of VX-809 was additive to either GRK5 or LRRK1 KD.

To more clearly describe the participation of hit kinases on ERQC checkpoints, we examined the combined effect of siRNA KD on the rescue of p.Phe508del-CFTR revertants (p.Phe508del-p.Gly550Glu, p.Phe508del-p.Arg1070Trp, p.Phe508del-4RK) or the DD/AA-CFTR traffic variant (Figure S6). Kinase KDs produced a complex response in the p.Phe508del-p.Gly550Glu and p.Phe508del-p.Arg1070Trp revertants, which target the first ERQC checkpoint. Knocking down LRRK1 or STYK1 did not significantly affect band C levels in most situations, either alone or in the presence of correctors, indicating that these KDs may act at the level of this folding checkpoint. However, STYK1 KD was additive to corrector treatment of p.Phe508del-p.Arg1070Trp-CFTR, suggesting targeting of the ICL4/NBD1 interface.^8^^,^^40^ DGKG KD was additive to VX-661 but the effect was lost in the presence of VX-445. TPK1 and DGKG KD were mostly additive to VX-661. Rescue by GRK5 KD was lost in the presence of correctors. However, VX-445 had a deleterious effect on band C when knocking down GRK5, TPK1, and DGKG on p.Phe508del-p.Gly550Glu-CFTR (Figure S6A), suggesting a common or a competing MoA at the NBD1:NBD2 pocket.^14^^,^^41^ The p.Phe508del-4RK-CFTR revertant was additive to all kinase KDs, alone or in the presence of correctors, except for TPK1 and GRK5, which produced unchanged (TPK1) or lower (GRK5) band C levels versus the drug alone. The DD/AA traffic variant was not affected.

Finally, we determined the specificity of hit kinases by assessing CFTR processing when performing kinase KDs in non-p.Phe508del-CFTR class II variants: p.Arg560Ser (legacy: R560S), p.Gly85Glu (legacy: G550E), and p.Asn1303Lys (legacy: N1303K) (Figure S7). No rescue was observed, even in combination with CFTR folding correctors, indicating that the five kinases specifically regulate p.Phe508del-CFTR traffic.

### GRK5 activity regulates p.Phe508del-CFTR PM localization and function

We considered the overall results obtained for the five hit kinases and selected GRK5 (G-protein-coupled receptor kinase 5) for additional mechanistic studies, as KD of this kinase yielded the best p.Phe508del-CFTR rescue performance in most assays, including patch-clamp and WB. The likely overlap with the pathway targeted by VX-445 is potentially useful to describe its MoA regarding CFTR regulation. GRK5 has also been proposed as a therapeutic target for cardiovascular disorders,^42^ making it an attractive candidate for drug repurposing. Furthermore, GRK5 is a regulator of endogenous CFTR activation in the airway epithelium^43^ through β_2_ adrenergic receptor (β_2_AR) signaling, acting at the level of receptor internalization, signal termination, and cell desensitization.^44^ Recently, the first GRK5-specific inhibitor was reported: CCG-273463 (also known as 9g).^45^ This 2-bromoacetylamido compound covalently binds GRK5 at low concentrations (IC_50_ = 8.6 nM) and with 1400-fold selectivity against the closely related paralog GRK2, thus being instrumental for complementing and validating the siRNA-based data.

**Figure 7:**
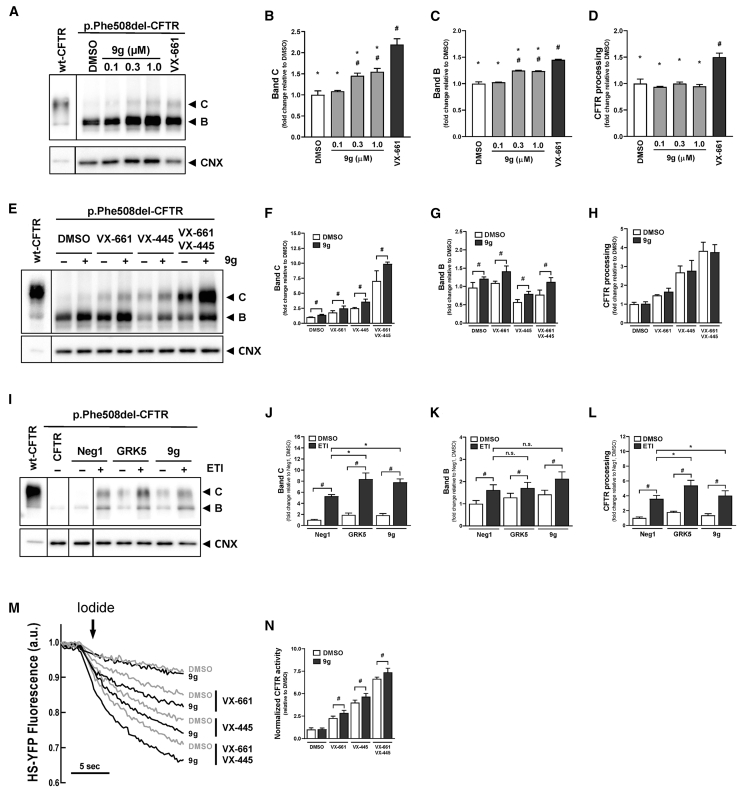
GRK5 inhibition with 9g rescues p.Phe508del-CFTR traffic and function (A) CFBE cells stably expressing p.Phe508del-CFTR were incubated with increasing doses of 9g or VX-661 3 μM and analyzed with WB. Densitometric quantification of band C (B), band B (C) and CFTR processing [C/(B + C)] (D). Significant band C rescue is observed at 0.3 and 1.0 μM of 9g. Processing is not affected due to proportional band B increases. Symbols indicate statistical significance from DMSO (“#”) or VX-661 (“∗”) lanes (*p* < 0.05, one-way ANOVA followed by Dunnett’s post-hoc test, *n* = 3 biological replicates). (E) Additivity of 9g (1 μM) to CFTR correctors VX-661 (5 μM), VX-445 (3 μM), and VX-661 + VX-445 and densitometric quantification of band C (F), band B (G), and processing (H). 9g is additive to all corrector treatments regarding bands C and B (#*p* < 0.05 double-tailed unpaired *t* test, *n* = 4 biological replicates). (I) Additivity of 9g (1 μM) to the triple combination of compounds that comprise the highly effective CFTR modulator therapy: VX-661 (5 μM), VX-445 (3 μM), and VX-770 (3 μM) and densitometric quantification of band C (J), band B (K), and processing (L). The modulator cocktail significantly rescues p.Phe508del-CFTR on all WB measurements. Furthermore, GRK5 inhibition with siRNA (GRK5) or 9g is additive to ETI treatment regarding band C and CFTR processing. Symbols indicate statistical significance from matching DMSO (“#”, *p* < 0.05, double-tailed unpaired *t* test, *n* = 4 biological replicates) or ETI-alone experiments (“∗”, *p* < 0.05, one-way ANOVA followed by Dunnett’s post-hoc test, *n* = 4 biological replicates). (M) HS-YFP quenching of CFBE cells expressing p.Phe508del-CFTR and incubated with 9g (1 μM) and/or VX-661 (5 μM), VX-445 (3 μM), and VX-661 + VX-445. (N) Quenching rate at the moment of iodide addition. 9g is additive to all corrector treatments regarding p.Phe508del-CFTR functional rescue (#*p* < 0.05 double-tailed unpaired *t* test, *n* = 4 biological replicates). All plot values are mean ± SD. Gel lanes were reordered and juxtaposed for presentation consistency and clarity.

To determine whether p.Phe508del-CFTR rescue could be obtained by inhibiting GRK5 with 9g, we started by performing a dose-response WB assay. CFBE cells expressing p.Phe508del-CFTR were incubated with 9g (0–1 μM) for 24 h, and band densitometry was compared with treatment with VX-661 (Figure 7A). Band C levels were the criterion used in the analysis of WB data because those are what really matters in terms of therapeutic benefit for people with CF, i.e., total amount of functional CFTR at the plasma membrane. We observed statistically significant band C rescue at 0.3 and 1.0 μM (50% of VX-661; Figure 7B), albeit without improved CFTR processing efficiency, due to a proportional increase in band B (Figures 7C and 7D). Higher 9g concentrations resulted in cellular toxicity and could not be used. Therefore, 1 μM was selected as the 9g concentration for further assays. To ascertain whether 9g is additive to CFTR correctors, we incubated CFBE cells expressing p.Phe508del-CFTR with 1 μM 9g in the presence and absence of VX-661, VX-445, or VX-661 plus VX-445. WB analysis (Figures 7E–7H) showed additivity of 9g to all corrector treatments, indicating a distinct MoA. Similarly, we performed HS-YFP quenching studies that also showed additivity in all conditions (Figures 7I and 7J).

To benchmark the effectiveness of p.Phe508del-CFTR rescue through GRK5 inhibition against the state-of-the-art highly effective modulator combination (VX-445 plus VX-661 plus VX-770 or elexacaftor plus tezacaftor plus ivacaftor, ETI), we combined the modulator cocktail with either genetic or chemical GRK5 inhibition and investigated band C levels (Figures 7I–7L). Band C rescue is higher when using ETI (5.3-fold) than with either VX-809 (∼2.8-fold over the control; Figure 5B) or VX-661 (3.7-fold; Figure 5F) in isolation. However, the combination VX-661 plus VX-445—which omits VX-770—yielded the highest extent of p.Phe508del-CFTR rescue: 7.0 (Figure 7F). Regardless, band C rescue by CFTR modulators is always further enhanced when applying genetic or chemical GRK5 inhibition (Figure 7). This can be illustrated by a rescue extent of 7.8-fold under ETI (Figure 7J) and 9.9-fold under VX-661 plus VX-445 (Figure 7F), when 9g is used to inhibit GRK5. These results not only validated the p.Phe508del-CFTR rescue through GRK5 inhibition—since genetic and chemical inhibition with a drug-like molecule yield equivalent results—but also supported the hypothesis that GRK5 inhibition and current CFTR modulators rescue p.Phe508del-CFTR by distinct mechanisms.

### GRK5 inhibition maximizes p.Phe508del-CFTR functional rescue in primary airway cells

To validate the biomedical relevance of pharmacological inhibition of GRK5 as a potential CFTR restoration strategy, we assessed its effects on primary human bronchial epithelial (pHBE) cells from individuals with CF. We noticed that our batch of in-house synthesized 9g had limited long-term stability and did not meet chemical quality standards by the time we had established polarized pHBE cell cultures for Ussing chamber analyses. To overcome this limitation, we resorted to CCG-273441 (also known as 9j), an analogue of 9g, which inhibits GRK5 with equivalent performance (IC_50, 9g_ = 8.6 nM, IC_50, 9j_ = 3.8 nM; selectivity: IC_50, GRK2_/IC_50, GRK5_ = 1400 for 9g, IC_50, GRK2_/IC_50, GRK5_ = 1300 for 9j)^45^ and can be obtained commercially (Figure S8A). In CFBE cells expressing p.Phe508del-CFTR, 9j is trophic at low concentrations, boosting cellular metabolism and proliferation above the DMSO control (Figure S8B). 9j rescued CFTR processing and ionic conductance in a manner additive to the VX-661 plus VX-445 combination (Figures S8C–S8H), reaching significance after 48 h exposure to 300 nM 9j. At these conditions, cell viability was found to be 74%. No significant rescue was observed in the absence of modulators.

**Figure 8:**
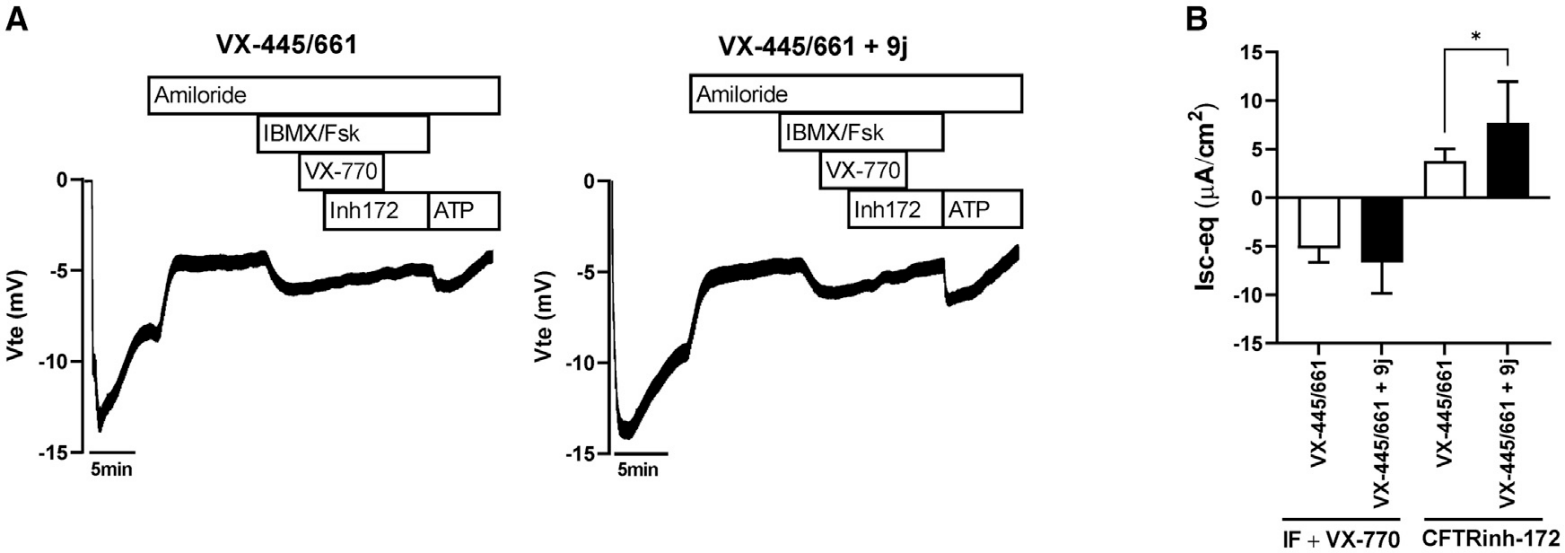
Effect of 9j on CFTR function in primary CF human bronchial epithelial (pHBE) cells (A) Representative Ussing chamber recordings of chloride transport measured as transepithelial voltage (Vte).Vte and transepithelial resistance (Rte) were recorded and used to calculate the equivalent short-circuit current (Isc-eq) using Ohm’s law: Isc-eq = Vte/Rte, as previously reported.^49^ Fully differentiated pHBE cells homozygous for p.Phe508del were pre-treated with a combination of 3 μM VX445 and 5 μM 661 for 48 h and additionally with DMSO (vehicle) or 500 nM 9j in the last 24 h of assay. Amiloride (ENaC channel inhibitor) was added apically, followed by a combination of IBMX and Fsk (cAMP-elevating agents), VX-770 (CFTR potentiator), and Inh172 (CFTRinh-172, CFTR specific inhibitor). (B) Summary of calculated equivalent short-circuit currents (Isc-eq) induced by IBMX/Fsk + VX-770 and inhibited by Inh172. Values are represented as mean ± SD (*n* = 7 biological replicates). ∗ represents significance (*p* value <0.05, unpaired *t* test).

The functional effect of 9j was assessed by measuring CFTR-mediated chloride (Cl^−^) transport in Ussing chamber using CF pHBE cells homozygous for p.Phe508del. This model was more tolerant to 9j and required slightly higher assay concentrations. Fully differentiated cells were pre-treated with VX-445/661 for 48 h, with 9j (500 nM) being added in the last 24 h of the assay. Ussing chamber tracings (Figure 8A) show a negative transepithelial voltage (V_te_) deflection upon IBMX/Fsk and VX-770 stimulation in both VX-445/661- and VX-445/661/9j-treated cells, indicating the activation of Cl^−^ secretion. The application of the specific CFTR inhibitor (CFTRinh-172), to confirm the induced ion transport is indeed CFTR-mediated, resulted in the reversal of the voltage direction in both conditions. When comparing the calculated equivalent short-circuit currents (Isc-eq) as presented in Figure 8B, there is a trend toward an enhanced Cl^−^ Isc-eq when 9j is combined with VX-445/661 compared to the treatment with VX-445/661 alone. Focusing on CFTR-mediated current, i.e., the Isc-eq inhibited by CFTRinh-172,^46^^,^^47^^,^^48^ this was found to be significantly higher for VX-445/661/9j-treated cells in comparison to cells treated with VX-445/661 alone, implying a higher CFTR-specific Cl^−^ transport in the presence of 9j. These results further confirm an additive effect of GRK5 inhibition to VX-445/661 by promoting CFTR rescue/trafficking to the cell surface and thus increasing CFTR function in primary CF airway epithelial cells.

## Discussion

We describe here an siRNA-based HCS campaign aimed at identifying genes whose KD enhances p.Phe508del-CFTR PM levels. Given the residual amount of PM p.Phe508del-CFTR, a major challenge in this work was the establishment of imaging and image analysis protocols that could record such baseline levels (low intensity and, therefore, relatively noisy) and their modulation by siRNA or drugs. Validation of HCS results by orthogonal assays demonstrated the robustness of the screening method. After siRNA screening of nearly half of the human genome, selecting hit genes, and re-screening them with different siRNAs, we confirmed that 35 genes are key inhibitors of p.Phe508del-CFTR traffic, as their KD enhances the PM localization of this CFTR variant. This stringent criterion equates to a 15% hit validation rate, somewhat lower than the 30% usually reported.^33^^,^^34^^,^^50^ This can be explained by low siRNA efficiency or specificity and sub-optimal transfection efficiency (Table S5). As we demonstrate, this does not imply that hits identified in the primary screen that were not confirmed in the secondary screen should be excluded as playing a role in p.Phe508del-CFTR traffic, as demonstrated for GRK5, our top p.Phe508del-CFTR regulator that was a hit recovered from the primary screen, with sub-optimal scores in the secondary screen.

Through a combination of WB validation experiments, gene expression analysis, and ERQC classification, we identified 53 genes whose KD showed F508deI-CFTR rescue in HCS or WB assays. The KD of the various hit genes had mixed effects on the sensitivity of CFTR to ERQC checkpoints, and it is therefore likely that they act by distinct MoAs. We noticed that besides affecting CFTR traffic and processing (e.g., WB band C), several hit siRNAs also affect the amount of immature p.Phe508del-CFTR (band B). In almost all cases we did not detect variations in CFTR mRNA expression (Figure S9A) and thus attribute the regulation of band B amount to post-translational events. Among the hit genes that significantly increased levels of p.Phe508-CFTR at the PM (and band C) with the highest scores were five kinases (TPK1, LRRK1, STYK1, GRK5, and DGKG), which we selected for additional validation because of their potential druggability. The unique property of GRK5 of simultaneously being an ENaC inhibitor^33^ is desirable for reversing CF symptoms in the clinic because the loss of Cl^−^ secretion via CFTR due to CF-causing variants is accompanied by Na^+^ hyperabsorption via ENaC, which exacerbates airway dehydration.^51^^,^^52^

To ascertain the biomedical relevance of inhibiting these kinases to achieve p.Phe508del-CFTR rescue, we performed WB and patch-clamp assays in combination with treatment with clinical CFTR correctors VX-809 and VX-661. Data from WB show that p.Phe508del-CFTR rescue by GRK5 and LRRK1 KD was additive to that of corrector treatment, suggesting independent MoAs. The TPK1 KD may compete with corrector rescue mechanisms, as a decreased rescue was observed in the presence of corrector drugs. Rescue of p.Phe508del-CFTR by STYK1 and DGKG KD was not additive to corrector treatment, thus suggesting that they target the same mechanisms. In patch-clamp experiments, the most significant rescue of p.Phe508del-CFTR function was achieved by GRK5 and LRRK1 KD, especially when combined with VX-809. We found that KD of the hit kinases was selective for p.Phe508del-CFTR versus other class II variants and that some of them could act through the first (Hsp70-mediated) ERQC checkpoint.

Altogether, we identified GRK5 as a new regulator of p.Phe508del-CFTR traffic and potentially relevant therapeutic target when inhibited, namely through pharmacological molecules 9g or 9j. This family of compounds is the first to selectively inhibit GRK5 vs. e.g., GRK2. Initially, inhibition was thought to derive from irreversible covalent binding to the active site^45^ but crystallographic data have since shown that binding is non-covalent.^53^ Low micromolar concentrations of 9g achieved significant rescue of p.Phe508del-CFTR traffic and function, which was additive to VX-809, VX-661, VX-661 plus VX-445, and the combination VX-770 plus VX-661 plus VX-445 (ETI), suggesting a potential added benefit. In this work, the highest extent of band C rescue occurred when combining 9g with VX-661 and VX-445 (9.9-fold over control). Rescue was lower under ETI (7.8-fold), which we attribute to the well-documented inhibitory effect of VX-770 on p.Phe508del-CFTR rescue when used in a chronic setting together with either VX-809,^54^^,^^55^ VX-661,^55^ or VX-664 plus VX-445.^56^

Despite being able to rescue band C, and additive with folding correctors toward processing (WB) and functional (HS-YFP) rescue, 9g or 9j alone were ineffective in rescuing p.Phe580del-CFTR function (Figures 7M and 7N). We interpret this observation as these inhibitors acting as correctors of p.Phe508del-CFTR trafficking but not of its gating defect. Based on WB data, 9j may effectively work as a “CFTR amplifier,” i.e., by exposing more CFTR molecules to modulator correction. We believe the additivity (i.e., distinct MoA) with CFTR correctors derives from the fact that the latter modulators bind specific pockets in the CFTR polypeptide,^13^ whereas GRK5 inhibitors are expected to act in an indirect manner, likely through modulation of CFTR molecular interactions. Regardless, the GRK5 inhibitor phenotype is always statistically significant when used in combination with p.Phe508del-CFTR folding correctors. Primary cell studies required 9j, a more potent—and perhaps more stable—GRK5 inhibitor than 9g. Again, we found that the inhibition of GRK5 in pHBE cells was fully additive to folding correctors in the restoration of p.Phe508del-CFTR PM levels and its chloride conductance.

**Figure 9:**
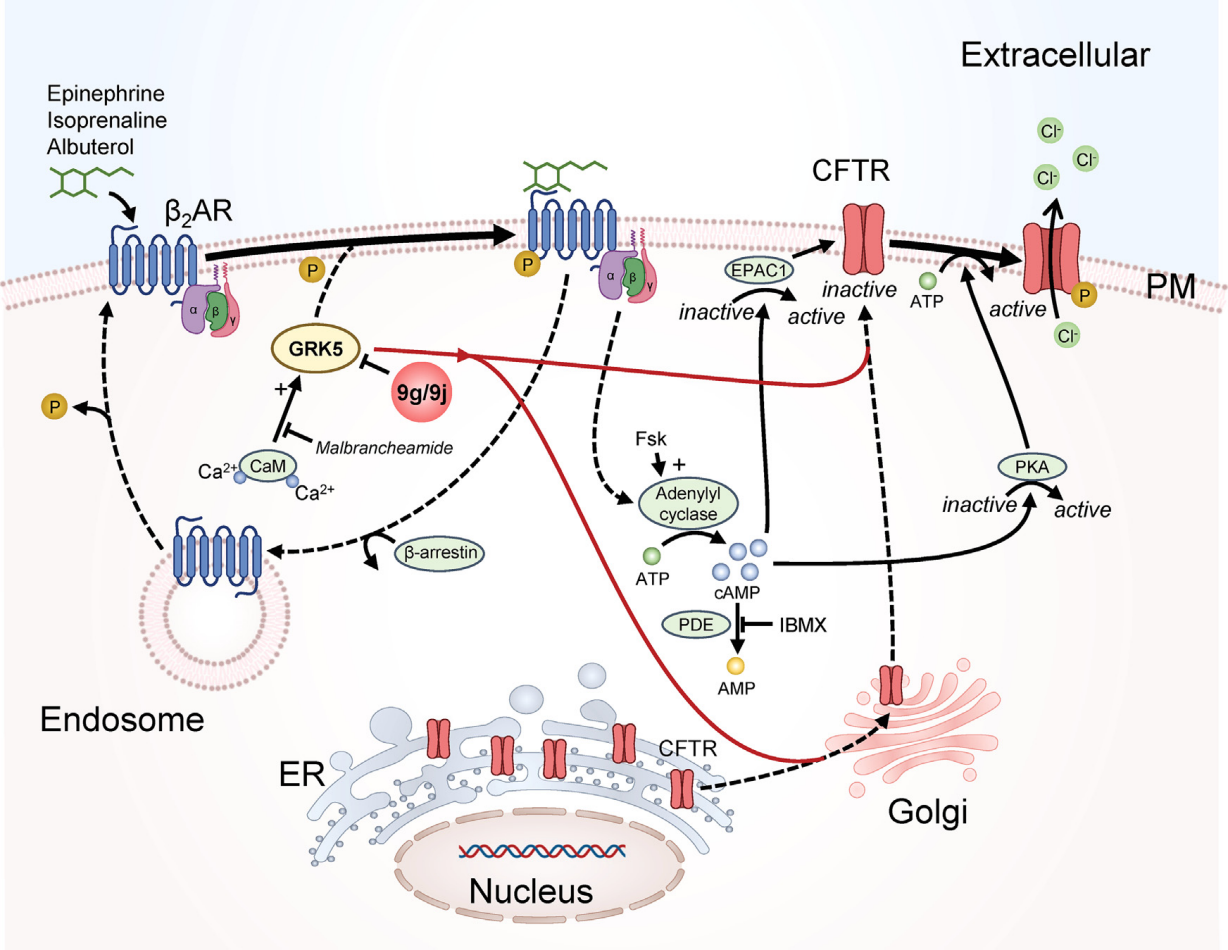
Model for p.Phe508del-CFTR rescue via GRK5 inhibition GRK5 is part of the machinery that activates CFTR endogenously, in response to β_2_-adrenergic receptor (β_2_AR) ligands such as epinephrine, isoprenaline, or albuterol. Ligand binding is transduced intracellularly by the hetero-trimeric G proteins associated with β_2_AR, which activate adenylyl cyclase. The resulting cAMP pool increase activates CFTR by (1) PKA activation, which phosphorylates CFTR’s R and initiates the gating cycle that opens the channel and (2) EPAC1-dependent stabilization of CFTR at the PM. Adenylyl cyclase can be artificially activated with forskolin (Fsk). The cAMP pool is depleted by phosphodiesterases (PDEs), which can be inhibited with 3-isobutyl-1-methylxanthine (IBMX). GRK5 phosphorylates activated β_2_AR, which then becomes substrate for β-arrestin and is endocytosed to interrupt receptor signaling. Endocytic vesicles are recycled to the PM to restore cell sensitization. GRK5 kinase activity is promoted by Ca^2+^-calmodulin (CaM). GRK5 inhibition with siRNAs, 9g, or 9j releases p.Phe508del-CFTR from the ER, increases the steady state at the PM, and yields higher CFTR-dependent Cl^−^ conductance, both in airway cell lines and primary cells. The link between GRK5 and p.Phe508del-CFTR ER release is not completely clear but given that GRK5 is not a CFTR interactor and its inhibition is additive to VX-661, a direct GRK5-CFTR mechanism is unlikely. Adapted from refs.^57^^,^^58^

The most likely MoA by which GRK5 inhibition rescues p.Phe508del-CFTR is through the well-established pathway of endogenous activation through β_2_AR^57^^,^^58^ (Figure 9). β_2_AR is a G-protein-coupled receptor (GPCR), and binding of extracellular β_2_AR agonists (e.g., epinephrine) is transduced intracellularly through trimeric G proteins, which activate adenylyl cyclase, the enzyme that converts ATP into cyclic AMP (cAMP).^59^ Indeed, cAMP contributes to functionally activate CFTR through two independent pathways: activation of protein kinase A (PKA) that phosphorylates CFTR R domain (RD) as the first step in channel gating^60^ and EPAC1-dependent stabilization at the PM through tethering to NHERF1.^61^ Termination of the adrenergic signal is dependent on GPCR kinases (GRKs). Humans express seven GRK isoforms (GRK1-7)^62^ that selectively bind the cytoplasmic face of ligand-bound—i.e., activated—GPCRs to phosphorylate Ser/Thr residues.^63^^,^^64^ Phosphorylated GPCRs become high-affinity targets for arrestins, which shield the cytoplasmic face of the receptor, preclude further G-protein binding and activation, and induce receptor endocytosis. Eventually, receptors are recycled to the PM to restore cell sensitization. The most well-studied GRKs are GRK2 and GRK5, two important therapeutic targets, as their inhibition can prevent heart failure and hypertrophic cardiomyopathy.^65^

Although it is still unclear how GRK5 inhibition releases p.Phe508del-CFTR from the ER, available CFTR interactomes seem to rule out a direct GRK5-CFTR interaction. Thus, the existence of additional mediators is most likely since no ER functions have yet been ascribed to GRK5. Interestingly, β_2_AR has been identified in a macromolecular complex tethering CFTR to the PM via NHERF1 through PDZ-based interactions,^43^ even though GRK5 involvement was not probed. Additionally, β_2_AR stimulation in airway epithelial cells enhances CFTR activity.^43^ Despite several reports relating GRK5 expression levels with neurodegenerative, cardiovascular, and metabolic diseases,^66^ we have not found significant alterations in GRK5 mRNA when comparing cells expressing wt- or p.Phe508del-CFTR (Figure S8B) or across DMSO versus 9g or ETI. We interpret this observation as the regulation of CFTR traffic and intracellular localization through GRK5 being due to dysregulated signaling events and not altered GRK5 expression. It is also unclear whether the possible impact on the cellular cAMP pool may be part of the GRK5 MoA. Endogenously, GRK5 is activated by interaction with Ca^2+^-calmodulin, a process that can be inhibited with malbrancheamide.^67^ This contribution was not assessed in this work because that would be a target upstream GRK5.

Our results identify GRK5 as a putative novel therapeutic target for CF, which can be inhibited with the selective inhibitors 9g or 9j to maximize p.Phe508del-CFTR rescue by current approved corrector drugs, including the highly effective modulator therapy combining ETI. The additivity of 9g/9j with CFTR modulators points to an independent MoA and a potential new pharmacological maneuver to augment CFTR function in the majority of individuals with CF. The low or sub-micromolar range where we observed rescue is at the same range as CFTR clinical correctors, indicating good potency. Available data on GRK5 and β_2_AR may provide a useful framework to further examine additional potential non-PM functions of GRK5 and the mechanistic connection between GRK5 inhibition and CFTR traffic regulation through this new druggable target.

### Limitations of the study

Some of the limitations of our study stem from the RNAi HCS methodology. Our screening library only covered about half of the human genome. This not only limits the overlap (and the ability to compare our results) with previous works but also raises the possibility that we may have missed to detect relevant CFTR regulator genes. Despite being a highly relevant CF model system, the cellular assay we used for the HCS assay might not feature all *in vivo* CFTR regulatory mechanisms and, therefore, not enable discovering all CFTR traffic regulator genes. siRNA-based assays—especially high-throughput assays, where transfection conditions cannot be optimized for all targets—commonly feature an inherently incomplete gene silencing, which is also the case for our study. This effect is likely to reduce the sensitivity to detect weaker or redundant CFTR regulators and can be overcome by shRNA- nor CRISPR/Cas9-based assays. It is possible that 9g can also be used effectively in the laboratory at incubation times other than the one we used (24 h). Finally, 9j dose-response was not exhaustively probed in pHBE epithelial cells, and there might exist experimental conditions that enhance CFTR rescue further than herein reported.

## Resource availability

### Lead contact

Further information and requests for resources and reagents should be directed to and will be fulfilled by the lead contact, Margarida D. Amaral (mdamaral@ciencias.ulisboa.pt).

### Materials availability

This study did not generate new unique reagents.

### Data and code availability

• Primary and secondary high content screening data have been deposited at Zenodo and are publicly available as of the date of publication. DOIs are listed in the key resources table.
• All original code has been deposited at Zenodo and is publicly available as of the date of publication. DOIs are listed in the key resources table.
• Any additional information required to reanalyze the data reported in this paper is available from the lead contact upon request.
• CellProfiler project files, shading correction files, and output files are available at https://doi.org/10.5281/zenodo.6617740. HCS data analysis scripts are available as reproducible R markdown files at the same URL. Interactome processing code is available at https://doi.org/10.5281/zenodo.6646236.

## Acknowledgments

Work supported by UIDB/04046/2020 (https://doi.org/10.54499/UIDB/04046/2020) and UIDP/04046/2020 (https://doi.org/10.54499/UIDP/04046/2020) Center grants from FCT, Portugal (to BioISI); UIDB/00100/2020 and UIDP/00100/2020 (to C.Q.E.) from FCT—Fundação para a Ciência e a Tecnologia, Portugal and research grants (to M.D.A.): TargetScreen2-FP6-2005-LH-7-037365 (European Union); PTDC/SAU-GMG/122299/2010 (10.13039/501100001871FCT, Portugal); Ref. 7207534 (CFF, USA) and (to H.M.B.) NewKinCF 2022.03453.PTDC (https://doi.org/10.54499/2022.03453.PTDC, FCT, Portugal). M.C.P. was recipient of a fellowship (SFRH/PD/BD/114393/2016) from BioSys PhD programme PD/00065/2012, from FCT, Portugal. H.M.B. held a Junior Researcher Contract from FCT (https://doi.org/10.54499/DL57/2016/CP1479/CT0012). M.L.P. is a recipient of the 2018 Gilead Sciences Research Scholars for Cystic Fibrosis. V.C. was recipient of a research contract funded by the PTSense research grant (AMARAL19G0) from CFF, USA (to M.D.A.). HCS was performed at the EMBL Advanced Light Microscopy Facility and Faculty of Sciences of the University of Lisboa Microscopy Facility, a node of the Portuguese Platform of BioImaging (PPBI), PPBI-POCI-01-0145-FEDER-022122 from FCT.

The authors are grateful to Dr. Nicoletta Pedemonte for providing the primary bronchial epithelial cells for this study.

## Author contributions

Conceptualization, H.M.B., M.L.P., M.C.P., B.N., C.M., K.K., R.P., and M.D.A. Methodology, H.M.B., M.L.P., M.C.P., V.C., C.T., and M.D.A. Software, H.M.B. and C.T. Validation, H.M.B., M.L.P., M.C.P., V.R., M.F.C., and M.D.A. Formal analysis, H.M.B., M.L.P., M.C.P., V.R., M.F.C., J.O., and L.A.C. Investigation, H.M.B., M.L.P., M.C.P., V.R., I.P., M.F.C., L.A.C., V.C., and J.O. Resources, H.M.B., V.C., K.K., R.P., and M.D.A. Data curation, H.M.B. Writing—original draft, H.M.B. and M.D.A. Writing—review and editing, all authors. Visualization, H.M.B., M.L.P., M.C.P., V.R., and M.F.C. Supervision, H.M.B., I.P., B.N., C.T., C.M., K.K., R.P., and M.D.A. Funding acquisition, M.D.A. and H.M.B.

## Declaration of interests

H.M.B., M.L.P., and M.D.A. are inventors in the international patent PCT/IB2023/051813 filed by the Faculty of Sciences of the University of Lisboa at the World Intellectual Property Organization that protects targeting the GRK5 or LRRK1 genes or proteins to rescue p.Phe508del-CFTR.

## STAR★Methods

### Key resources table

**Table:** 

REAGENT or RESOURCE	SOURCE	IDENTIFIER
**Antibodies**		

Mouse monoclonal anti-FLAG (clone M2)	Sigma-Aldrich	Cat#F1804; RRID: AB_262044
Donkey anti-mouse Alexa Fluor 647	Invitrogen	Cat#A31571; RRID: AB_162542
Mouse monoclonal anti-CFTR	CFF Therapeutics	Cat#596; RRID: AB_2923486
Mouse monoclonal anti-calnexin (clone 37)	BD Transduction Laboratories	Cat#610523; RRID: AB_397883

**Chemicals, peptides, and recombinant proteins**		

Puromycin	Sigma-Aldrich	Cat#P8833
Blasticidin	InvivoGen	Cat#ant-bl
G418	Sigma-Aldrich	Cat#A1720
Doxycycline	Sigma-Aldrich	Cat#9891
Forskolin	Sigma-Aldrich	Cat#F6886
IBMX	Sigma-Aldrich	Cat# I5879
CFTRinh-172	MedChemExpress	Cat#HY-16671
Genistein	Sigma-Aldrich	Cat#G6649
VX-445	Selleckchem	Cat#S8851
VX-661	Selleckchem	Cat#S7059
VX-770	Selleckchem	Cat#S1144
VX-809	Selleckchem	Cat#S1565
Hoechst 33342	Sigma Aldrich	Cat#B2261
9g (CCG-273463)	This work, synthesized as in Rowlands et al.^45^	N/A
9j (CCG-273441)	MedChemExpress	Cat#HY-47573
Resazurin	Sigma-Aldrich	Cat#R7017

**Critical commercial assays**		

SsoFast EvaGreen	Bio-Rad	Cat#1725201

**Deposited data**		

Image and data analysis of the primary and secondary siRNA screens	This paper	https://doi.org/10.5281/zenodo.6617740
CFTR interactomes	This paper plus Pankow et al.,^28^ Canato et al.,^29^ Reilly et al.,^30^ Tomati et al.,^31^ Dang et al.,^32^ Almaça et al.,^33^ Simpson et al.^34^	https://doi.org/10.5281/zenodo.6646236

**Experimental models: Cell lines**		

CFBE41o- mCherry-Flag-wt-CFTR	Botelho et al.^26^	https://doi.org/10.1038/srep09038
CFBE41o- mCherry-Flag-p.Phe508del-CFTR	Botelho et al.^26^	https://doi.org/10.1038/srep09038
CFBE41o- mCherry-Flag-p.Phe508del-4RK	Canato et al.^29^	https://doi.org/10.1007/s00018-018-2896-7
CFBE41o- mCherry-Flag-p.Phe508del-p.Arg1070Trp-CFTR	Canato et al.^29^	https://doi.org/10.1007/s00018-018-2896-7
CFBE41o- mCherry-Flag-p.Phe508del-p.Gly550Glu-CFTR	Canato et al.^29^	https://doi.org/10.1007/s00018-018-2896-7
CFBE41o- mCherry-Flag- DD/AA-CFTR	Canato et al.^29^	https://doi.org/10.1007/s00018-018-2896-7
CFBE41o- YFP-H148Q/I152L wt-CFTR	Laboratory of Nicoletta Pedemonte	https://doi.org/10.1152/ajpcell.00507.2010
CFBE41o- YFP-H148Q/I152L p.Phe508del-CFTR	Laboratory of Nicoletta Pedemonte	https://doi.org/10.1152/ajpcell.00507.2010
CFBE41o- wt-CFTR	Laboratory of John P. Clancy	https://doi.org/10.1113/jphysiol.2005.096669
CFBE41o- p.Phe508del-CFTR	Laboratory of John P. Clancy	https://doi.org/10.1113/jphysiol.2005.096669

**Experimental models: Organisms/strains**		

Primary human bronchial epithelial cells	Laboratory of Nicoletta Pedemonte	N/A

**Oligonucleotides**		

Ambion Silencer Human Extended Druggable Silencer siRNA library	Ambion	N/A
Secondary and classification siRNA libraries (Silencer Select)	Ambion	This paper
siRNA (Silencer Select) Negative control Antisense (5’ -> 3′): UUACGUCGUCG CGUCGUUAtt	Ambion	Cat#4390843
siRNA (Silencer Select) targeting DGKG Antisense (5’ -> 3′): UUCUGUAUAUUAG UAUCUGcg	Ambion	Cat#s3918
siRNA (Silencer Select) targeting GRK5 Antisense (5’ -> 3′): UUUCAGAUCUCGGU AGACGgt	Ambion	Cat#s6087
siRNA (Silencer Select) targeting LRRK1 Antisense (5’ -> 3′): UCAUAUACAAUGCG AGGCCtg	Ambion	Cat#s36138
siRNA (Silencer Select) targeting STYK1 Antisense (5’ -> 3′): UAGUAACCAACAA AGUUGGga	Ambion	Cat#s30824
siRNA (Silencer Select) targeting TPK1 Antisense (5’ -> 3′): AAAAUAGUUGUCC AAAGGCtg	Ambion	Cat#s25692
PCR primer sequences	Harvard primerbank	https://pga.mgh.harvard.edu/primerbank

**Software and algorithms**		

CellProfiler	Carpenter et al.^68^	http://cellprofiler.org
R	R Core Team	https://cran.r-project.org/
R scripts	This paper	https://doi.org/10.5281/zenodo.6617740
ImageLab	Bio-Rad	https://www.bio-rad.com/product/image-lab-software
PULSE	HEKA	N/A
Chart	AD Instruments	N/A
Panther	Laboratory of Paul D. Thomas	http://www.pantherdb.org
GraphPad Prism	GraphPad	https://www.graphpad.com/scientific-software/prism

**Other**		

Multidrop™ Combi	ThermoFisher	Cat#5840300
Leica DMI6000 B	Leica Microsystems	N/A
Infinite F200 Pro	Tecan	N/A
EPC 7 patch clamp amplifier	List Medical Electronics	N/A
LIH1600 interface	HEKA	N/A
Victor 3V	PerkinElmer	N/A

### Experimental model and study participant details

#### Bronchial epithelial cell lines

mCherry-Flag-CFTR with Tet-ON expression in CFBE41o^−^ cells were described elsewhere,^26^^,^^69^ representing wt- and p.Phe508del-CFTR,^26^ p.Phe508del revertant variants p.Phe508del-4RK (p.Arg29Lys, p.Arg516Lys, p.Arg555Lys, p.Arg766Lys), p.Phe508del-p.Arg1070Trp-CFTR, p.Phe508del-p.Gly550Glu-CFTR, and DD/AA-CFTR.^29^ Cells were grown in DMEM containing 4.5 g/L Glucose and L-Glutamine (Lonza, #12-604F) supplemented with 10% FBS (Gibco #10106), 2 μg/mL puromycin (Sigma-Aldrich, #P8833) and 10 μg/mL Blasticidin (InvivoGen, #ant-bl) at 37°C in 5% CO_2_. CFBE41o^−^ cells constitutively co-expressing HS-YFP (YFP-H148Q/I152L) and wt- or p.Phe508del-CFTR^70^ were a kind gift from Dr Nicoletta Pedemonte (IRCCS Istituto G. Gaslini, Genoa, Italy). Cells were cultured in MEM supplemented with 10% FBS, 2 mM L-glutamine, 2 μg/mL puromycin and 200 μM G418 (Sigma-Aldrich, # A1720) at 37°C in 5% CO_2_. CFBE41o^−^ cells constitutively expressing wt- or p.Phe508del-CFTR^71^ were grown in DMEM, 10% FBS and 2 μg/mL puromycin at 37°C in 5% CO_2_.

#### Primary human bronchial epithelial cells

Primary human bronchial epithelial cells (pHBE), obtained from one p.Phe508del/p.Phe508del CF individual, were kindly provided by Dr. Nicoletta Pedemonte, IRCCS Istituto Giannina Gaslini, Genova, Italy. pHBE cells were cultured and expanded in Pneumacult ExPlus media according to the manufacturer’s protocol (StemCell Technologies) and seeded on collagen-type I-coated (30 μg/mL, Advanced Biomatrix, 5005) porous inserts (Transwell, Corning, USA). After reaching confluency, cells were differentiated in Pneumacult-ALI media (StemCell Technologies) for 28 to 42 days in air-liquid interface (ALI) conditions. Then, CFTR-mediated Cl^−^ transport was assessed in cells exposed to CFTR modulators (VX-445 plus VX-661), with and without GRK5 inhibitor 9j (see “ussing chamber recordings” below). A total of 7 inserts were assayed for each condition.

#### Ethics statement

The collection of bronchial epithelial cells (supported by Fondazione per la Ricerca sulla Fibrosi Cistica through the “Servizio Colture Primarie”) and their study to investigate the mechanisms of transepithelial ion transport were specifically approved (on 8 July 2018) by the Ethics Committee of the IRCCS Istituto Giannina Gaslini following the guidelines of the Italian Ministry of Health (registration number: ANTECER, 042-09/07/2018). Each patient provided informed consent to the study using a form that was also approved by the Ethics Committee.

### Method details

#### Chemicals

VX-445 (#S8851), VX-661 (#S7059), VX-770 (#S1144) and VX-809 (#S1565) were purchased from Selleckchem, USA. Amiloride, 3-isobutyl-1-methylxanthine (IBMX, #I5879), Forskolin (#F6886) were purchased from Sigma-Aldrich, USA. CFTRinh-172 (#HY-16671) and CCG-273441 (9j, #HY-47573) were purchased from MedChemExpress, USA. 9g (CCG-273463) was synthesized in-house, as described below.

#### siRNA libraries

The primary screen used the Ambion Silencer Human Extended Druggable Silencer siRNA library laid out in 384 well plates.^27^ Only siRNAs targeting a single protein-coding gene (as of Ensembl78) were considered. Plate coating with siRNAs (described in Botelho et al.^26^) failed in 8 plate layouts, which were discarded. Primary screen hits were re-screened with a cherry-picked Ambion Silencer Select siRNA library (2 siRNAs per gene target) using the same coating protocol. Validation assays (WB and HS-YFP quenching) used siRNAs from the secondary screening library and the same coating protocol.

#### CFTR traffic assay: primary screen

To establish an assay compatible with siRNA-based gene KD and small molecule treatments, a total experiment time of 72 h was selected, a consensus time in other studies.^6^^,^^72^ In the primary screen CFTR intracellular localization was assessed in CFBE cells expressing the inducible mCherry-Flag-p.Phe508del-CFTR traffic reporter and subjected to siRNA KDs. Cells were split 24h before the start of the experiment to synchronize them in exponential growing phase and maximize transfection efficiency. The experiment was initiated by seeding cells in transfection-ready microscopy-grade multi-well plates.^73^ At the moment of CFTR induction the siRNA target gene is – at least partially – absent. Each well was seeded with 2,500 cells using a peristaltic pump (ThermoFisher Multidrop Combi) in the absence of antibiotics. Expression of mCherry-Flag-p.Phe508del-CFTR was induced with 1 μg/mL doxycycline (Dox, Sigma-Aldrich, #9891) starting at 24 h after seeding and continuing for 48 h, to allow for sufficient construct expression (mCherry fluorescence). The positive controls were wells containing a non-targeting siRNA (Scrambled), induced with 3 μM VX-809 and 0.1% FBS.

After the 72h assay time, immunofluorescence was performed to detect extracellular Flag tags in non-permeabilized cells.^26^^,^^27^ Briefly, cells were washed once in ice-cold PBS supplemented with 0.7 mM CaCl_2_ and 1.1 mM MgCl_2_ (PBS^++^), incubated 1 h with an anti-Flag antibody (1:500, Sigma-Aldrich #F1804), washed three times with ice-cold PBS^++^, incubated 20 min with 3% paraformaldehyde (PFA), washed three times with room temperature PBS^++^, incubated 1 h with an anti-mouse Alexa Fluor 647 conjugated secondary antibody (1:500, Invitrogen #A31571), washed three times with PBS^++^, incubated with Hoechst 33342 (200 ng/mL, Sigma #B2261) for 1 to 6 h, washed with PBS^++^ and stored at 4°C until imaging. Antibodies were solubilized in PBS^++^ containing 1% BSA (Aldrich #A9647). PFA and Hoechst were solubilized in PBS^++^.

#### CFTR traffic assay: Secondary screen

The 227 primary screen hit genes were re-screened with 2 additional siRNAs using the traffic assay described for the primary screen and CFBE cells expressing the mCherry-Flag-p.Phe508del-CFTR reporter. siRNAs with improved stability and specificity (Ambion Silencer Select) and different sequences than the ones in the primary screen were used (see siRNA libraries).

#### CFTR traffic assay: ERQC checkpoints classification screen

Classification of the primary screen hits in terms of their effect on the ERQC checkpoints which retain p.Phe508del-CFTR was pursued by re-screening the primary screen hits using the reporter cell lines expressing wt-CFTR, p.Phe508del-CFTR genetic revertants (4RK, p.Gly550Glu or p.Arg1070Trp) as well as the DD/AA variant.

#### Image acquisition

The imaging workflow was based on preliminary experiments performed on an Olympus ScanR widefield system.^26^ Imaging was performed at room temperature with a Leica DMI6000 B inverted epifluorescence microscope equipped with a metal halide light source (EL6000), a 10x NA 0.40 HC PL APO objective, a Hamamatsu Orca-Flash4.0 CMOS camera and Leica Microsystems filter cubes suitable for imaging the fluorophores used in the traffic assay: Hoechst 33342 (model A: excitation: 34 BP0–380; dichroic mirror 400; emission filter LP 425), mCherry (model N2.1: excitation 51 BP5–560; dichroic mirror 580; emission LP 590) and Alexa Fluor 647 (custom: excitation: 63 BP0–660; emission LP 670). The microscope was controlled by the Leica MatrixScreener software, using software autofocus and 4 image fields per well. Images were metadata annotated and organized with the R package htmrenamer^74^ and stored as 16 bit OME-TIFF.

#### Image analysis

Image quantification was performed with CellProfiler (http://cellprofiler.org/),^68^ including dark field/flat frame background correction, cell segmentation, fluorescence integration and basic quality control (exclusion of cells undergoing mitosis, apoptosis or containing a significant amount of saturated pixels). Three cell-based features were extracted: integrated mCherry fluorescence signal (total CFTR expression), integrated Alexa Fluor 647 fluorescence (amount of CFTR molecules inserted into the PM) and the ratio of the integrated fluorescence signals from Alexa Fluor 647 and mCherry (A647/mCherry, CFTR traffic efficiency). Hit thresholding was based on the PM signal. The secondary and classification library plates contained 5–8 wells with siRNA targeting CFTR, as transfection control. Only plates where mCherry fluorescence was lost (i.e., abrogation of CFTR expression) in the majority of cells were scored.

#### Data analysis – primary screen

The PM CFTR phenotype generated by each siRNA was scored through R scripts. The algorithm takes as input image-based features (e.g., metadata, focus score, background fluorescence) and cell-based features (e.g., the image they belong to, metadata, fluorescence intensities, traffic efficiency ratio) generated by image analysis with CellProfiler and computes a modified *Z* score for the PM fluorescence intensity and traffic efficiency ratio produced by each siRNA. The algorithm has been implemented in the R programming language and has the following steps: (i) Import image and cell features; (ii) Quality Control: exclude data from unapproved plates (bad sample preparation), wells (wells with less than 100 cells), images (out-of-focus or high background images) and cells (aberrant morphology, fluorescence saturation or low CFTR expression); (iii) Well Summary: take the PM fluorescence intensity and traffic efficiency values for all cells in a well and compute their respective medians; (iv) Normalization: convert each well summary value into a score. Due to the lack of reliable non-targeting siRNAs in the primary screen library, the negative control measurements for each well were provided by neighboring ones, as defined by a 5 × 5 matrix centered at the target well. For external wells, which do not have 24 neighbors, missing values were dropped. Data was normalized with a modified *Z* score defined by:

$$Modified\;Z-score=\frac{x-Median\left({Neighbors}_{{5\times 5,plate}_{i}}\right)}{sd\left({Neighbors}_{{5\times 5,plate}_{i}}\right)}$$

where *x* is the well summary value, *sd* is the standard deviation and ${Neighbors}_{{5\times 5,plate}_{i}}$ is the ensemble of negative control values; *v)* Plate summary: compute the median of the normalized PM staining and traffic efficiency values for all wells which have the same siRNA treatment in the same plate; *vi)* Treatment summary: take the plate summary values and compute their median across replicate plates. The median-summarized values are the ones we report in this publication.

#### Data analysis – Secondary and classification screens

In the secondary and classification libraries 10 to 18 wells in each 384 well plate contained a non-targeting siRNA (Neg1) as negative control. Data was analyzed as in the primary screen, with two differences: (i) also discarding plates with low transfection efficiency in wells containing CFTR siRNA; and (ii) using a standard *Z* score at the normalization step, using the average and standard deviation of wells containing the non-targeting Neg1 siRNA.

#### Halide-sensitive YFP fluorescence quenching

The assay followed an established protocol.^70^ For siRNA-based assays, CFBE cells co-expressing HS-YFP and wt- or p.Phe508del-CFTR were grown to confluency and split to 50%. On the following day, the experiment was initiated by seeding cells onto siRNA-coated 96 well plates using a peristaltic pump (10,000 cells/well, in antibiotic-free medium). 48 h later, VX-809 (3 μM) or VX-661 (5 μM) were added to selected wells containing Neg1 siRNA in reduced FBS (0.1%). 72 h after seeding (as in microscopy-based screens), cells were washed two times with PBS (containing 137 mM NaCl, 2.7 mM KCl, 8.1 mM Na_2_HPO_4_, 1.5 mM KH_2_PO_4_, 1 mM CaCl_2_, and 0.5 mM MgCl_2_) and stimulated for 30 min with PBS containing Forskolin (20 μM) and VX-770 (3 μM) at 37°C under atmospheric CO_2_. Each well was added 60 μL of the stimulation solution. After stimulation, each plate was transferred to a fluorescence plate reader equipped with fast injectors (Tecan Infinite F200 Pro) and YFP excitation (47 BP5–495 nm) and emission (52 BP3-458 nm) filters. Each assay consisted of a continuous 16 s fluorescence reading with 2 s before and 14 s after injection of 160 μL of a PBS where Cl^−^ had been replaced by I^−^ (final I^−^ concentration in the well: 100 mM). The pH of all PBS-containing solutions was rigorously set at pH 7.4 immediately before use to avoid artifacts in the fluorescence reading. Kinetic data was background subtracted and normalized to the pre-injection value. The final 14 s of the kinetics in each well were fitted with a single decay exponential curve and the derivative at the injection time (i.e., the quenching rate) was extracted from the curve parameters. The negative of the quenching rate was converted to *Z* score or fold change versus the negative control. For 9j assays, p.Phe508del-CFTR expressing cells were grown in MEM with 10% FBS and seeded at 18,500 cells/well. 9j and modulators were added 24 h later, in reduced FBS medium (1%) and were incubated for 48 h (replacing with fresh identical medium midway).

#### Western blotting (WB)

Assays used CFBE cells constitutively expressing wt-, p.Phe508del-, p.Phe508del-p.Gly550Glu-, p.Phe508del-p.Arg1070Trp-, p.Phe508del-4RK-, DD/AA-, p.Gly85Glu- or p.Asn1303Lys-CFTR. Confluent cell cultures were split to 50% confluency, to stimulate cell proliferation and enhance transfection efficiency. 24 h later, cells were seeded into 12-well plates which had been coated with siRNAs for reverse transfection (87,500 cells/well). The plate coating protocol was the same as for the microscopy screening assay. Each plate included a non-targeting Neg1 siRNA as negative control and a CFTR targeting siRNA as transfection control. Cells were grown for 48 h in the presence of siRNAs. When required, VX-809 (3 μM), VX-661 (5 μM), VX-445 (3 μM) or VX-770 (3 μM) were added in the last 24 h of siRNA contact – alone or in combination, as required – along with matching DMSO controls. 9j response assays used CFBE cells expressing p.Phe508del-CFTR seeded at a density of 110,000 cells/well in 24 well plates and grown in MEM media. 24h after seeding, cells were exposed to DMSO or VX-661 plus VX-445 in combination with 9j at several concentrations for 24 or 48 h. In 48 h incubation studies, the culture medium was replaced with fresh and identical medium at the incubation midpoint.

WB samples were prepared by lysing transfected cells with Laemmli sample buffer. The buffer contained no bromophenol and was supplemented with a protease inhibitor cocktail (Roche, Complete: one tablet per mL), benzonase and magnesium chloride. Cells were scrapped manually at 4°C and lysates were stored at −20°C until analysis. Following quantification, the same protein amount of each lysate was loaded on SDS-PAGE gels (7.5% or 10%), electrophoresed, blotted and probed with anti-CFTR (CFFT 596, 1:1000-1:3000) or anti-Calnexin (BD Transduction Laboratories #610523, 1:3000) antibodies. Membranes were developed with a luminescent enzymatic reaction. wt-CFTR samples had lower concentration to allow proper visualization of the much more abundant CFTR bands.

Images were quantified with Bio-Rad’s Image Lab software, with local background subtraction. Band densitometry was initially transformed into fold change versus the loading control. To compare across bands, results were reported as fold change versus p.Phe508del-CFTR-expressing cells transfected with the Neg1 siRNA without correctors.

#### Cell viability assay

To assess cell viability using a metabolic reporter, we used the resazurin reduction assay. CFBE cells expressing p.Phe508del-CFTR were cultured on MEM supplemented with 10% FBS and seeded on 96 well clear bottom plates (Greiner #655090), at a density of 18,500 cells/well. 24 h later, culture media was replaced MEM supplemented with 1% FBS and either DMSO or VX-661 (5 μM) plus VX-445 (3 μM) plus 9j (0–30 μM). Cells incubated for a total of 48 h, with compounds being refreshed at the incubation midpoint. After the incubation, 5 wells on each plate were exposed to 30% ethanol for 20 s, to provide a 0% viability control. Then, cells were incubated with 10 μg/mL resazurin (Sigma-Aldrich #R7017) on DMEM supplemented with 1% FBS for 1 h at 37°C. Fluorescence (excitation 531/40 nm; emission 595 nm) was measured on a Victor 3V plate reader (PerkinElmer). Viability was computed using the 0% (ethanol) and 100% (DMSO vehicle) controls. The 9j EC50 was determined through a logistic fit to the concentration-dependent data.Patch-clamp.

The p.Phe508del-CFTR chloride conductance was investigated in CFBE cells overexpressing p.Phe508del-CFTR and transfected with the following Ambion Silencer Select siRNAs against selected hit genes: siDGKG (s3918), siGRK5 (s6087), siLRRK1 (s36138), siSTYK1 (s30824) and siTPK1 (s25692). siRNA selection was based on WB experiments: each gene was knocked-down with two distinct siRNAs and the one producing the largest p.Phe508del-CFTR rescue was selected. The negative control siRNA was Neg1.

Preliminary control experiments were performed on cells expressing wt-CFTR. Cells grown on cover slips were mounted in a perfused bath on the stage of an inverted microscope (IM35, Zeiss) and kept at 37°C. The bath was perfused continuously with Ringer solution (mM: NaCl 145, KH_2_PO_4_ 0.4, K_2_HPO_4_ 1.6, D-glucose 5, MgCl_2_ 1, Ca-gluconate 1.3, pH 7.4) containing 50 nM TRAM-34 at about 8 mL/min. Patch-clamp experiments were performed in the fast whole-cell configuration. Patch pipettes had an input resistance of 2–4 MΩ, and whole cell currents were corrected for serial resistance. Patch pipettes were filled with an intracellular like solution containing (mM) KCl 30, K-gluconate 95, NaH_2_PO_4_ 1.2, Na_2_HPO_4_ 4.8, EGTA 1, Ca-gluconate 0.758, MgCl_2_ 1.034, D-glucose 5, ATP 3. pH was 7.2, the Ca^2+^ activity was 0.1 μM. According to the protocol we have previously established,^75^ the patch pipette is slightly hypotonic (270 mosmol/l when compared to the extracellular bath solution, which is 290 mosmol/l. Currents were recorded using a patch clamp amplifier (EPC 7, List Medical Electronics, Darmstadt, Germany), the LIH1600 interface and PULSE software (HEKA, Lambrecht, Germany) as well as Chart software (AD Instruments, Spechbach, Germany). In regular intervals, membrane voltages (Vc) were clamped in steps of 20 mV from −100 to +100 mV from holding potential of −100 mV. CFTR was stimulated with 25 μM Genistein and 2 μM Forskolin (Gen/Fsk). Current density was calculated by dividing whole-cell currents by cell capacitance. The calculated chloride equilibrium potential was −37.0 mV.

#### Ussing Chamber recordings

pHBE cells were treated with 3 μM VX-445 in combination with 5 μM VX-661 for 48h. DMSO or 500 nM 9j were added in the last 24h. CFTR function was assessed in Ussing Chamber as previously described by Awatade et al.^49^ The transepithelial voltage (Vte) and transepithelial resistance (Rte) were recorded and used to calculate the equivalent short-circuit current (Isc-eq) using Ohm’s law: Isc-eq = Vte/Rte. Experiments were performed by sequentially adding apically 20 μM amiloride, followed by 100 μM IBMX in combination with 2 μM Forskolin (Fsk), 10 μM VX-770, and 30 μM CFTRinh-172.

#### Quantitative RT-PCR

RNA was extracted (Trizol method—Invitrogen—or Macherey Nagel Nucleospin columns), quantified by Nanodrop spectrophotometry and digested with DNase I. Approximately 1 μg of total RNA was subjected to reverse transcription (RT) with random primers to produce cDNA (NZYTech, Lisboa, Portugal). cDNA was diluted to give a final reaction mixture concentration between 0.5 and 1.5 ng/μL. Each gene transcript was amplified in separate reactions by qRT-PCR using the SsoFast EvaGreen system (Bio-Rad, Hercules, CA), with primer sequences obtained from the Harvard primerbank (https://pga.mgh.harvard.edu/primerbank, Data S1). Melt curves were checked to confirm amplification of single products, and negative controls were confirmed to be free of nonspecific amplification at 40 cycles. Products were quantified using the ΔΔCT method, normalized using as a reference gene either CAP-1 (adenylate cyclase associated protein 1) or ACTB (β actin). The correct size of amplification products was verified by agarose gel electrophoresis.

#### Bioinformatic analyses

The scores from different CFTR variants obtained at the ERQC classification screen were compared with a heatmap coupled to hierarchical clustering, using a custom R script relying on the heatmaply package.^76^ Data was kept in the *Z* score scale and was not further normalized.

Statistical overrepresentation tests of the Gene Ontology annotation were performed on Panther (http://www.pantherdb.org),^77^ with Bonferroni correction. The complete term database was used.

#### Intersections of current data with other CFTR-related datasets

The following datasets were considered: the primary and confirmed hit genes enhancing p.Phe508del-CFTR traffic (this study), p.Phe508del-CFTR interactome in HBE41o^−^ cells,^28^ p.Phe508del-CFTR interactome in CFBE 410- cells,^29^^,^^30^ genes whose silencing significantly rescued p.Phe508del-CFTR activity,^31^ candidate modifier genes of CF lung disease,^32^ ENaC activating genes in A549 cells^33^ and protein secretion machinery in HeLa cells.^34^ Gene identification was based on Uniprot IDs.

#### Chemistry general remarks

All solvents were purchased from commercial sources and were dried according to standard methods. All chemicals and starting materials (2-oxoindoline-5-carboxylic acid, (S)-1-phenylethan-1-amine, HATU, 3,5-dimethyl-4-nitro-1H-pyrrole-2-carbaldehyde, bromoacetic acid, DIPEA, piperidine and zinc powder) were purchased from Fluorochem and Sigma-Aldrich. Reactions were monitored by TLC using 0.25 mm silica gel 60 F_254_ TLC plates purchased from Merck. Spots were visualized under ultraviolet light (254 nm).

^1^H, ^13^C APT NMR spectra were acquired on a Bruker Avance 400 spectrometer equipped with a 5 mm QNP probe operating at 400.16 MHz and 100.61 MHz for ^1^H and ^13^C respectively, at 293K. Spectra of all compounds were recorded using DMSO-*d*_*6*_ (99.9%; Eurisotop, UK). Spectroscopic data of all compounds was matched with the one previously reported.^45^ High-Resolution-Mass Spectrometry (HR-MS) and low-resolution Mass Spectrometry (MS) spectra were performed on an Impact II QTOF (Bruker, Bremen, Germany) mass spectrometer with an electrospray ionization source (ESI). The method consisted of direct infusions with MS/MS scans, in the positive and negative modes.

#### 9g synthetic procedure

**Scheme 1:**
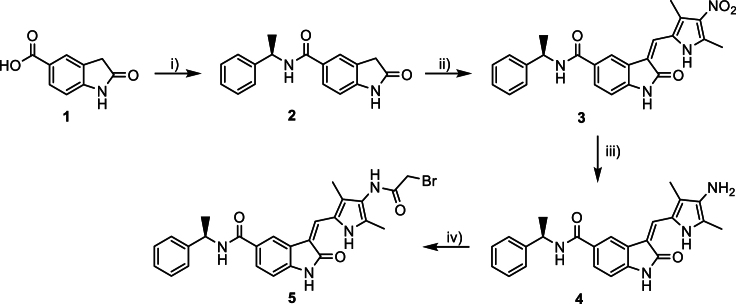
Synthetic route for 9g (i) (S)-1-phenylethylamine, HATU, DIPEA, DMF, rt, overnight, 84%; (ii) 3,5-dimethyl-4-nitro-1H-pyrrole-2-carbaldehyde, piperidine, EtOH, 95°C, 2 h, 87%;0 (iii) zinc powder, AcOH, EtOAc, EtOH, 50°C, 2 h, 79%; (iv) bromoacetic acid, HATU, DIPEA, DMF, rt, overnight, 55%.

The (R,Z)-3-((4-(2-bromoacetamido)-3,5-dimethyl-1H-pyrrol-2-yl)-methylene)-2-oxo-N-(1-phenylethyl)indoline-5-carboxamide (9g, 5) was synthesized employing slight modifications of the Rowlands et al. method.^45^ In the second step, reaction time was cut in half and the yield increased to 87%. In the last step of the synthesis, the reported coupling agent was DMTMM with a yield of 39%, while we used HATU which afforded the final compound (5) in 55% yield (Scheme 1). The NMR spectra of the final product is shown in Figure S10.

### Quantification and statistical analysis

Results are presented as mean or median ±SD or SEM, with the number of biological replicates indicated in the figure, figure legend or accompanying supplementary dataset. Hypothesis testing was performed by unpaired two-tailed Student’s *t*-tests (pairwise comparison), one-way ANOVA followed by Dunnett’s post-hoc test (multiple comparisons) or one sample *t*-test, as implemented in the GraphPad Prism software. Correlation analysis was performed on R. 0.05 significance was required.

## Supplemental information

Document S1. Figures S1–S10 and Tables S1–S5

Data S1. Primary screen scoring and hit genes

Data S2. Gene Ontology (GO) overrepresentation tests for primary screen gene hits

Data S3. Secondary screen scoring and hit genes

Data S4. RT-PCR results for the 53 genes that were assayed in western blot experiments We analyzed the 35 confirmed hit genes (secondary screen) and 18 additional genes selected among the siRNAs that scored slightly below the hit threshold in the confirmation screen. CT values for expression in control lung tissue and CFBE cells overexpressing wt-CFTR. All genes amplified a product with single melt peak. The sequences of the primers used for amplification are shown.

Data S5. Scoring of the functional assessment by the HS-YFP fluorescence quenching assay for the 53 confirmed hit genes KDs

Data S6. Classification screen scoring of the genes in the secondary siRNA library for their effects at ERQC checkpoints Quantification for the following CFTR variants: wt, p.Phe508del-4RK-CFTR, p.Phe508del-p.Gly550Glu-CFTR, p.Phe508del-p.Arg1070Trp-CFTR, DD/AA-CFTR.
