# Supplementary material for: Global functional genomics reveals GRK5 as a cystic fibrosis therapeutic target synergistic with current modulators
Source: iScience. 2025 Feb 1;28(3):111942. doi: 10.1016/j.isci.2025.111942 (PMC11876911; doi:10.1016/j.isci.2025.111942)
Supplement: Document S1. Figures S1–S10 and Tables S1–S5 [file mmc1.pdf]

iScience, Volume *28*

Supplemental information

Global functional genomics reveals GRK5 as a cystic fibrosis therapeutic target synergistic with current modulators

Hugo M. Botelho, Miquéias Lopes-Pacheco, Madalena C. Pinto, Violeta Railean, Ines Pankonien, Mariana F. Caleiro, Luka A. Clarke, Vasco Cachatra, Beate Neumann, Christian Tischer, Cristina Moiteiro, Jiraporn Ousingsawat, Karl Kunzelmann, Rainer Pepperkok, and Margarida D. Amaral

Supplemental Data

Table of Contents

Figure S1. High-content screening strategy, related to Figures 1, 2 and Methods.
Figure S2. Primary screen hit pathways, related to Figure 1.
Figure S3. Western blot analysis of the KD of primary screen genes which were not confirmed in the secondary rescreening step, related to Figure 1 and 3.
Figure S4. Mechanistic analysis of the 53 confirmed hit genes, related to Results (“Classification of traffic regulators”).
Figure S5. Whole cell currents measured in CFBE cell expressing wt-CFTR as a function of the Cl- concentration distribution, related to Figure 6.
Figure S6. Western blot analysis of the additivity of CFTR folding correctors on the glycosylation of DD/AA-CFTR and p.Phe508del-CFTR traffic revertants, related to Results (“Classification of traffic regulators”).
Figure S7. Western blot analysis of the additivity of CFTR folding correctors on the glycosylation of selected non-p.Phe508del CFTR class II variants, related to Results (“Classification of traffic regulators”).
Figure S8. Inhibition of GRK5 with CCG-273441 (also known as 9j) rescues p.Phe508del-CFTR processing and ionic conductance, related to Figure 8.
Figure S9. Expression of CFTR and GRK5 mRNA in CFBE cells, related to Figures 3 and 7.
Figure S10. NMR spectra for compound 9g, related to Methods (“9g Synthetic Procedure”).

Table S1. Confirmed hit genes and scores (secondary screen), related to Figures 1, 2 and Dataset S3.
Table S2. Effect of top hit genes on p.Phe508del-CFTR processing assessed by WB, related to Figure 3 and S3.
Table S3. Specificity analysis of the classification screen, related to Figure 1 and S4 and Dataset S6.
Table S4. ERQC analysis of the classification screen, related to Figure 1 and S4 and Dataset S6.
Table S5. Knock-down efficiency of hit kinase genes, related to Figure 4 and 6.

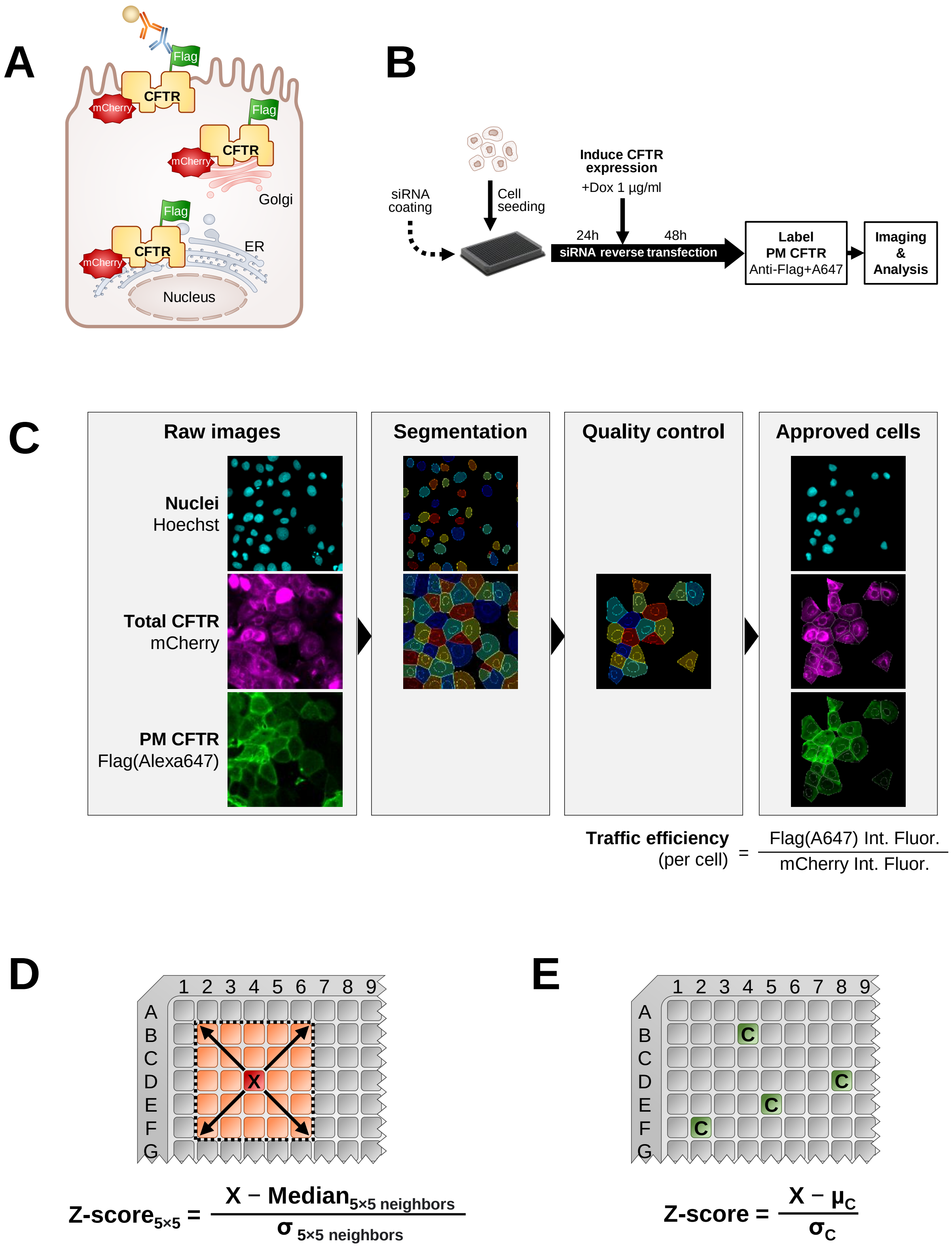

Figure S1. High-content screening strategy, related to Figures 1, 2 and Methods. (A) Topology of the mCherry-Flag-CFTR traffic reporter, expressed in CFBE cells under the control of a Tet-inducible promoter. PM CFTR can be immunolabeled in unpermeabilized cells. (B) Workflow of the CFTR traffic assay for siRNA screening. (C) Image quantification algorithm, showing the mCherry-Flag-wt-CFTR construct. Initially, nuclei are segmented and used as seeds to estimate the cytoplasm location. Then, cells touching the image border, missing CFTR expression or containing apoptotic nuclei or saturated pixels are excluded from analysis. Fluorescence is quantified in approved cells, in the mCherry (total CFTR expression) or Flag(A647) (PM CFTR) channels. Data from images which are either out-of-focus

or contain few cells are discarded (not shown). (D) Z-score calculation in the primary screen. The reference value for Z-score calculation is provided by the 5×5 well neighbourhood, when available. (E) Z-score calculation in the secondary, classification and 9g screens: classical control-based Z-score.

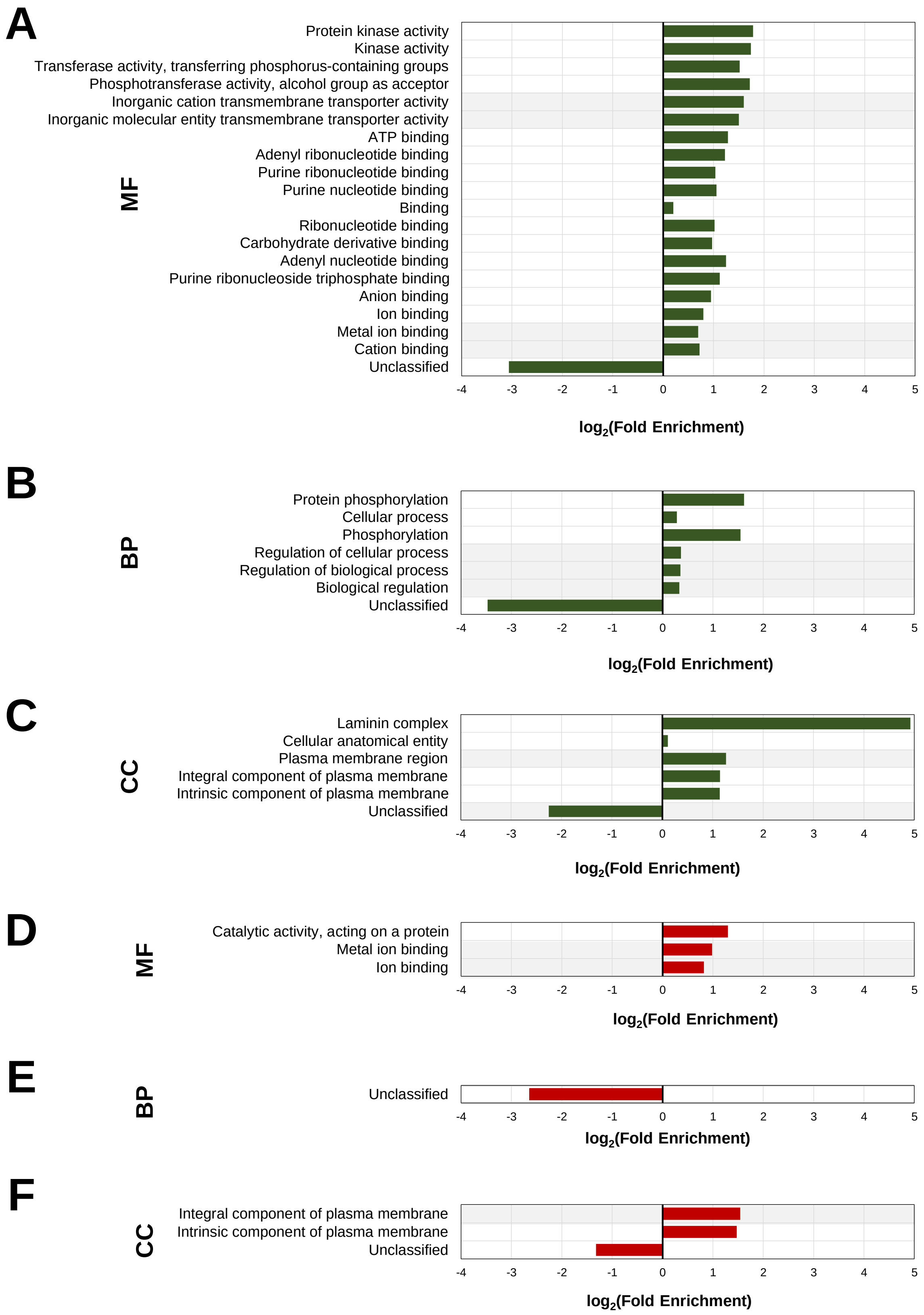

Figure S2. Primary screen hit pathways, related to Figure 1. Panther was used to assess the overrepresentation of GO annotation terms of enhancer genes hits: (A) molecular function, (B) biological process, (C) cellular compartment. Panther was used to assess the overrepresentation of GO annotation terms of inhibitor genes hits: (D) molecular function, (E) biological process, (F) cellular compartment.

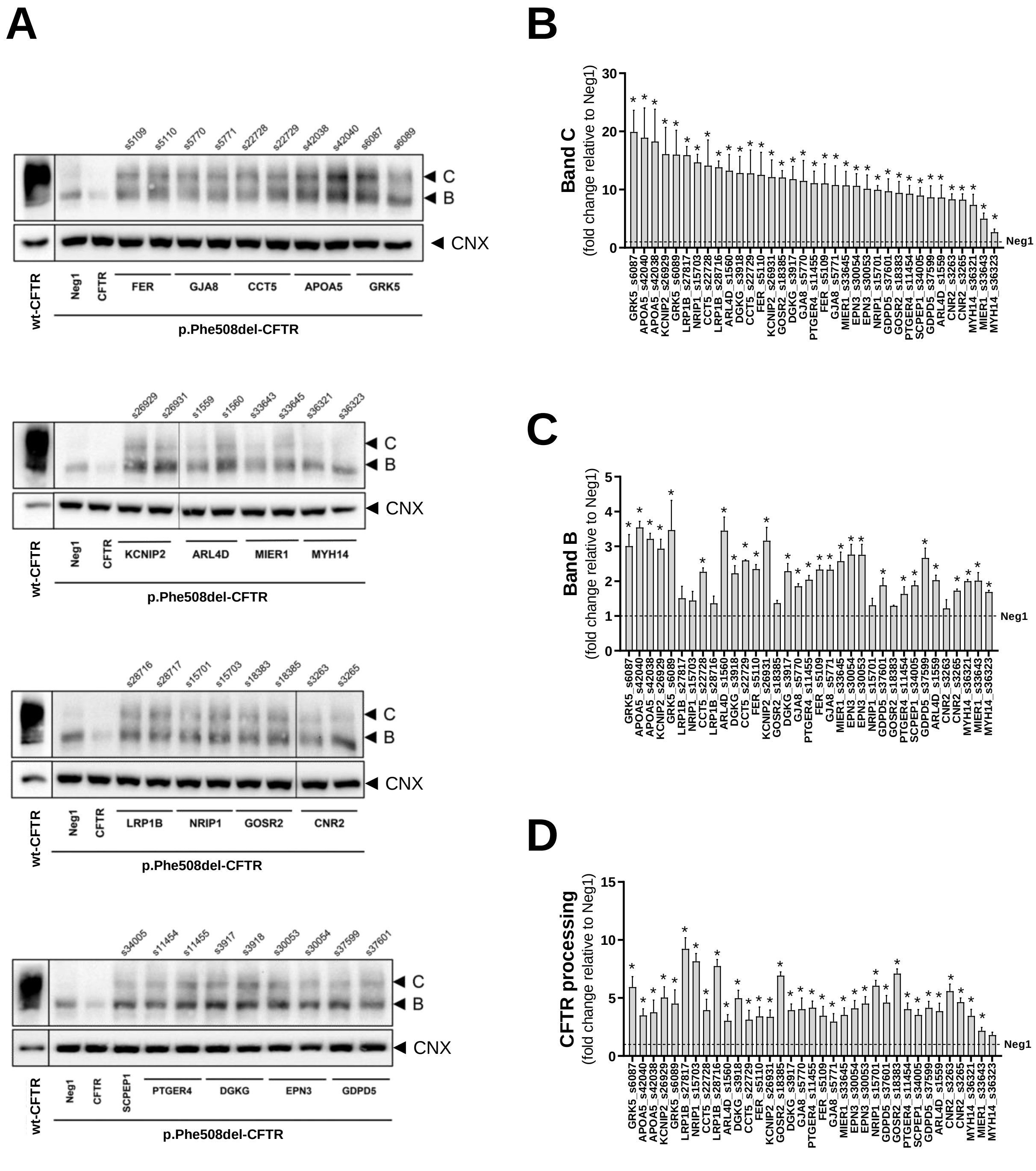

Figure S3. Western blot analysis of the KD of primary screen genes which were not confirmed in the secondary rescreening step, related to Figure 1 and 3. CFBE cells overexpressing p.Phe508del-CFTR were transfected with siRNAs targeting 18 high-scoring primary hits which were not confirmed in the HT microscopy assay. (A) CFTR detection in a WB membrane. wt-CFTR was included for reference. The localization of core glycosylated (band B) and fully glycosylated CFTR (band C) are shown. Quantification of band B (B), band C (C) as well as p.Phe508del-CFTR processing efficiency [C/(B+C)] (D). The baseline readout, given by cells treated with the Neg1 siRNA, is given as a dotted line. ´*´ indicates statistical significance from Neg1-treated ($p<0.05$, one-way ANOVA followed by Dunnett's post-hoc test, n=3 biological replicates). Plot values are mean ± SD. Gel lanes were reordered and juxtaposed for presentation consistency and clarity.

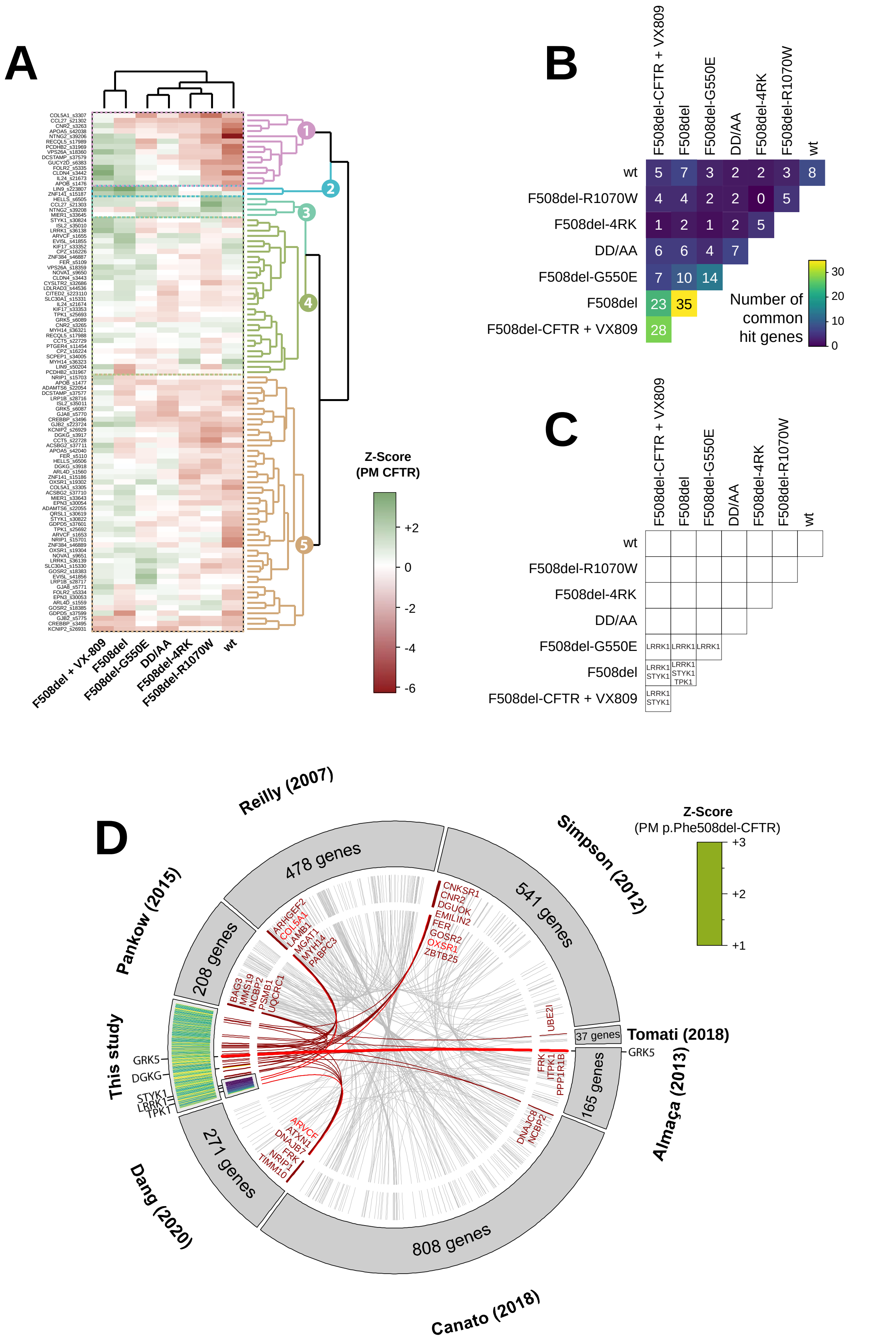

Figure S4. Mechanistic analysis of the 53 confirmed hit genes, related to Results ("Classification of traffic regulators"). (A) Hierarchical clustering of the classification screen results for the 53 genes whose KD was confirmed to rescue p.Phe508del-CFTR traffic (HCS) or processing (WB). Each line in

the y-axis is a single siRNA. The rows dendrogram highlights the 5 clusters mentioned in the results. For simplicity, single letter amino acid codes are used. (B) Number of common hit genes for the several CFTR variants. Legacy variant names are used for simplicity. (C) Simplified representation of A, showing only hit kinases. (D) Co-occurrence of genes in the p.Phe508del-CFTR traffic screens (this study) and other CFTR-related datasets: p.Phe508del-CFTR interactome in HBE410- cells (Pankow et al., 2015), p.Phe508del-CFTR interactome in CFBE41o- cells (Canato et al., 2018; Reilly et al., 2017), genes whose silencing significantly rescued p.Phe508del-CFTR activity (Tomati et al., 2018), candidate modifier genes of CF lung disease (Dang et al., 2020), ENaC activating genes in A549 cells (Almaça et al., 2013) and protein secretion machinery in HeLa cells (Simpson et al., 2012). For this study, the outer track represents the primary screen hits and the inner track represents the subset of 35 HCS confirmed genes. Each gene is represented as a line color coded for the Z-score. Inter-dataset comparisons were performed based on Uniprot IDs. Genes for which an updated Uniprot ID could not be found were discarded, explaining the discrepancy in the gene counts in the original publications and this figure. Common genes in published datasets are linked by thin gray arcs in the center of the plot. Primary screen hits are linked by thin dark red arcs. Hit genes conformed by HCS or WB are linked by thin bright red arcs. Hit kinase genes are linked by thick bright red arcs. Selected gene names are shown in corresponding red tones. GRK5 (an ENaC activating gene) is the only kinase reported in another study.

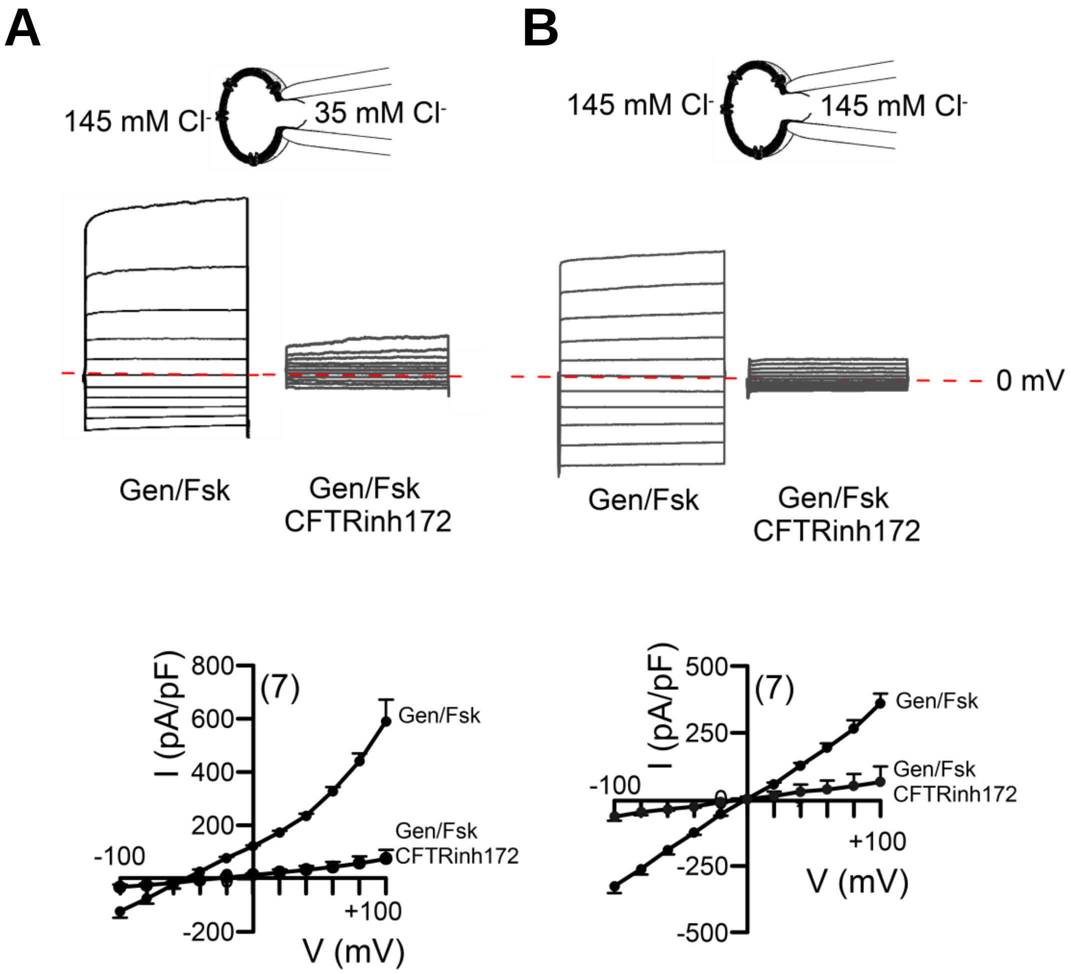

Figure S5. Whole cell currents measured in CFBE cell expressing wt-CFTR as a function of the Cl^- concentration distribution, related to Figure 6. (A) Our patch clamp experiments were performed under physiological conditions: 37 °C, perfused experimental bath, and in the presence of an intracellular (pipette) ion composition that come close to cellular conditions, with Ringer solution in the bath (about 145 mM Cl^-) and a cytosolic-like ion composition in the patch pipette filling solution (containing (mM) KCl 30, K-gluconate 95, NaH_2PO_4 1.2, Na_2HPO_4 4.8, EGTA 1, Ca-gluconate 0.758, $MgCl_2$ 1.034, D-glucose 5, ATP 3. pH was 7.2, and Ca^{2+} activity 0.1 µM). Under these conditions there is a Cl^- gradient from outside 145 mM to inside (patch pipette) 35 mM and currents are outwardly rectifying when CFTR is activated through stimulation with forskolin (2 µM) and genistein (25 µM). **(B)** Under symmetric Cl^- conditions, the activated CFTR current is symmetric and linear (no longer outwardly rectifying). Moreover, in the presence of additional $CFTR_{inh}172$ (20 µM), the activated current is strongly (95 %) inhibited under either condition, indicating activation of CFTR currents, with little contribution of other currents. Values are mean ± SD. Numbers within parenthesis are the replicate count.

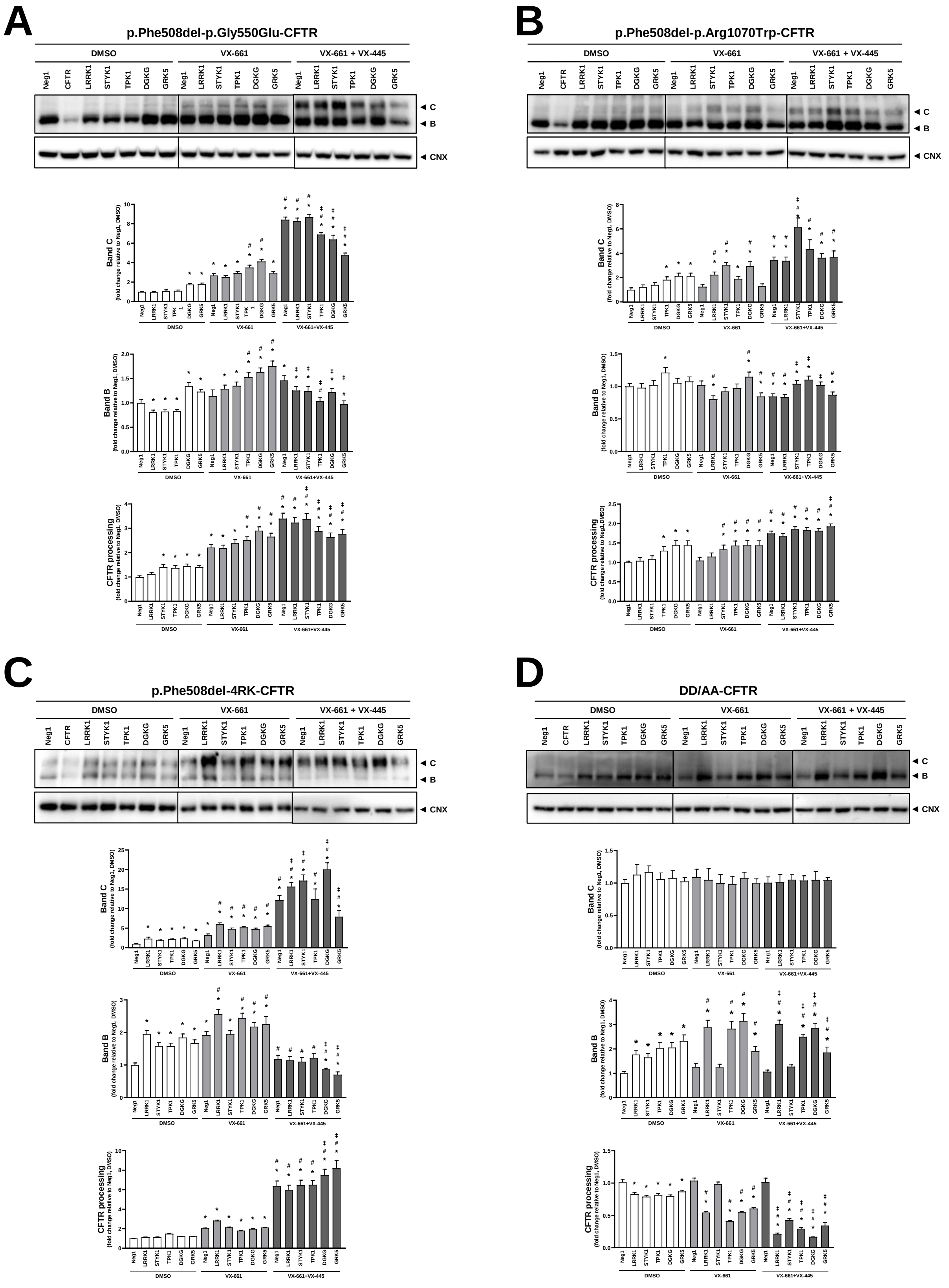

Figure S6. Western blot analysis of the additivity of CFTR folding correctors on the glycosylation of DD/AA-CFTR and p.Phe508del-CFTR traffic revertants, related to Results ("Classification of traffic regulators"). WB analysis of CFBE cells expressing p.Phe508del revertants p.Phe508del-p.Gly550Glu-CFTR (A), p.Phe508del-p.Arg1070Trp-CFTR (B), p.Phe508del-4RK-CFTR (C) or the DD/AA-CFTR variant (D). The quantification of CFTR's band B, C and processing [C/(B+C)] is shown. Symbols indicate statistic difference versus Neg1/DMSO- (´*´), Neg1/VX-445 (´#´) or Neg1/VX-664/VX-

445-treated cells (´‡´) ($p<0.05$, one-way ANOVA followed by Dunnett's post-hoc test. n=4 biological replicates, except for p.Phe508del-4RK-CFTR treated with VX-445, n=3). Plot values are mean ± SD. Gel lanes were reordered and juxtaposed for presentation consistency and clarity.

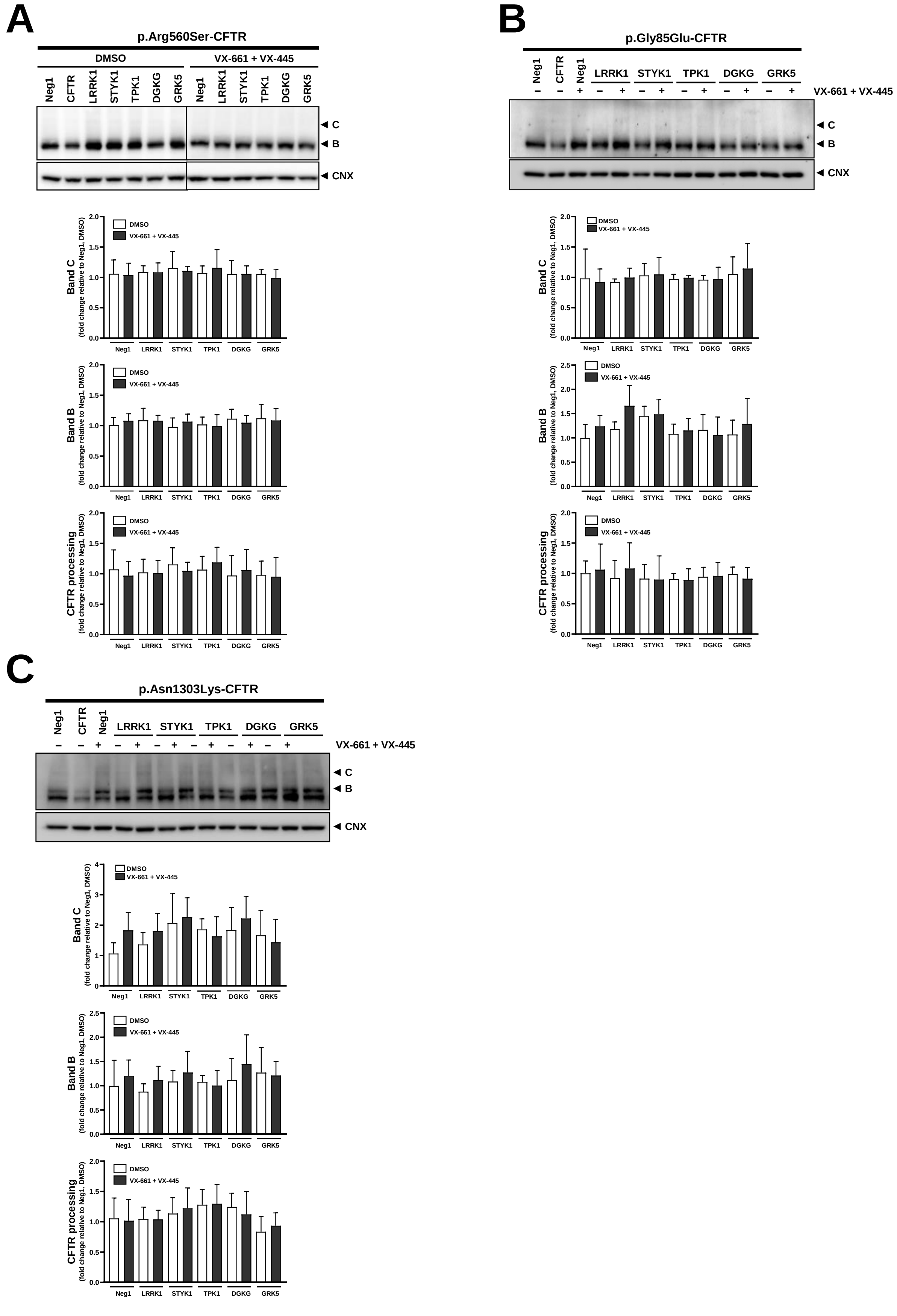

Figure S7. Western blot analysis of the additivity of CFTR folding correctors on the glycosylation of selected non-p.Phe508del CFTR class II variants, related to Results ("Classification of traffic regulators"). CFBE cells overexpressing p.Arg560Ser- (A), p.Gly85Glu- (B) or p.Asn1303Lys-CFTR

(C) were transfected with Neg1 or siRNAs targeting LRRK1, STYK1, TPK1, DGKG or GRK5, together with DMSO or a VX-661 (5 µM) plus VX-445 (3 µM) combination and the CFTR glycosylation pattern was analysed by WB. Representative membranes and quantification of band C, band B and processing efficiency, all normalized to the calnexin (CNX) loading control. $p > 0.05$ (unpaired *t*-test, VX-661/VX-445 versus corresponding DMSO, n=3 biological replicates). Plot values are mean ± SD. Gel lanes were reordered and juxtaposed for presentation consistency and clarity.

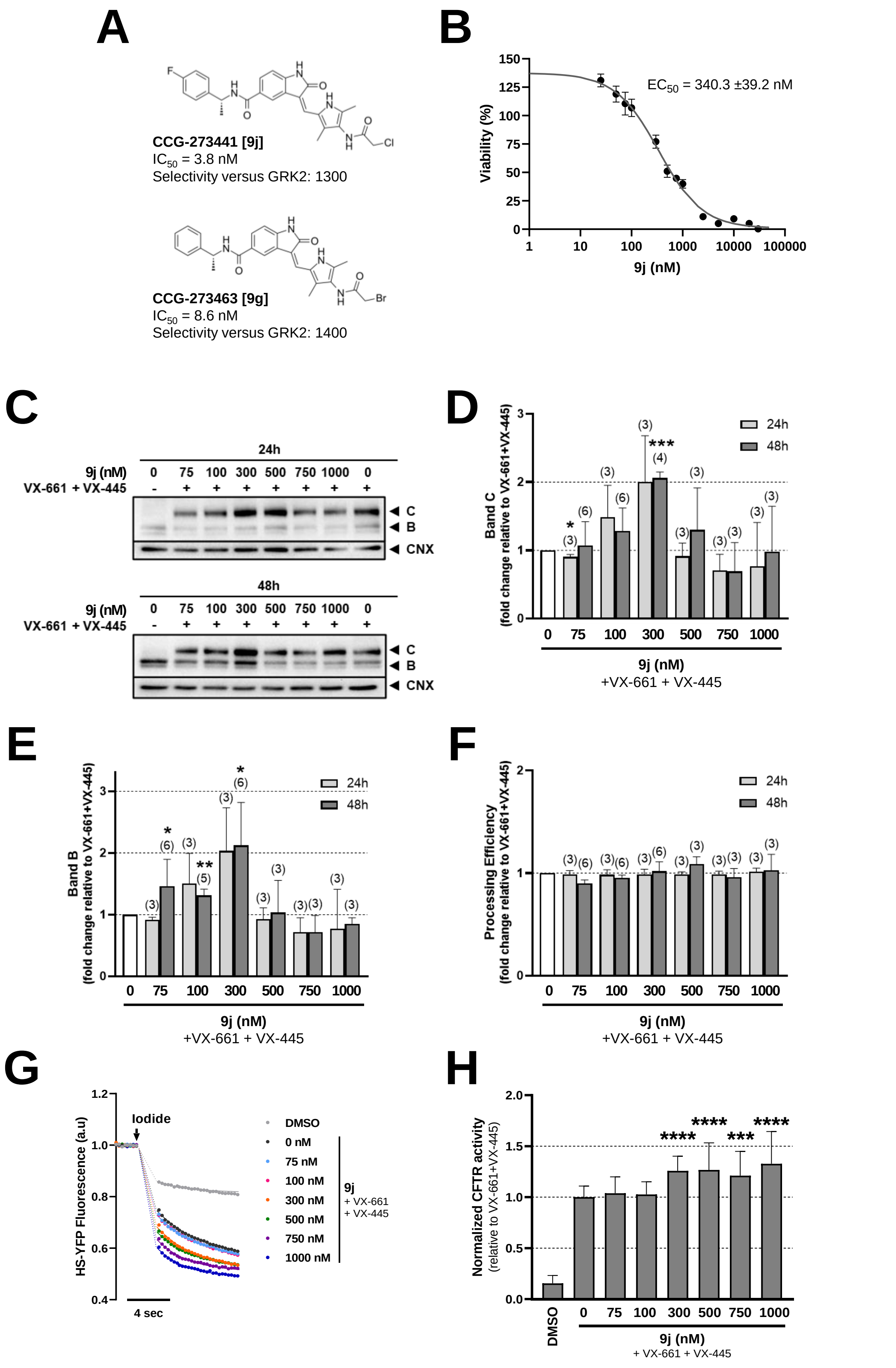

Figure S8. Inhibition of GRK5 with CCG-273441 (also known as 9j) rescues p.Phe508del-CFTR processing and ionic conductance, related to Figure 8. (A) 9j is a structural analogue of 9g, with equivalent chemical and biochemical properties, available from a quality-controlled commercial source

(see methods) and with stability suitable for several month-long rounds of assays. **(B)** Dose-response curve and EC_{50} for CFBE p.Phe508del-CFTR cells grown in the presence of 9j. Obtained with the resazurin reduction assay (n=3 biological replicates). **(C)** CFBE cells expressing p.Phe508del-CFTR were incubated with VX-661 (5 µM) and VX-445 (3 µM) in the absence or presence of 9j, for 24 or 48 h. Densitometric analysis revealed a significant rescue of band C after 48 h exposure to 300 nM 9j **(D)**. Given the concomitant increase in band B **(E)** without change in processing efficiency **(F)** (one sample *t*-test), we surmise that the mechanism of action is increasing the CFTR steady state concentration and, thereby, exposing more molecules to folding correction by modulators. Replicate numbers are shown above each bar. **(G)** Representative HS-YFP quenching curves obtained with CFBE cells co-expressing p.Phe508del-CFTR and HS-YFP after 48h treatment with DMSO, and VX-661 plus VX-445 alone and in combination with 9j. The arrow indicates iodide addition. Exponential fits and dashed lines (during the 2-second iodide addition period) were added to guide the eye. **(H)** CFTR activity quantification based on the HS-YFP quenching rate normalized to VX-661 and VX-445 combination. One-way ANOVA followed by Dunnett's post-hoc test, n=4 biological replicates. *, $p < 0.05$; **, $p < 0.01$; ***, $p < 0.001$. All values are mean ± SD.

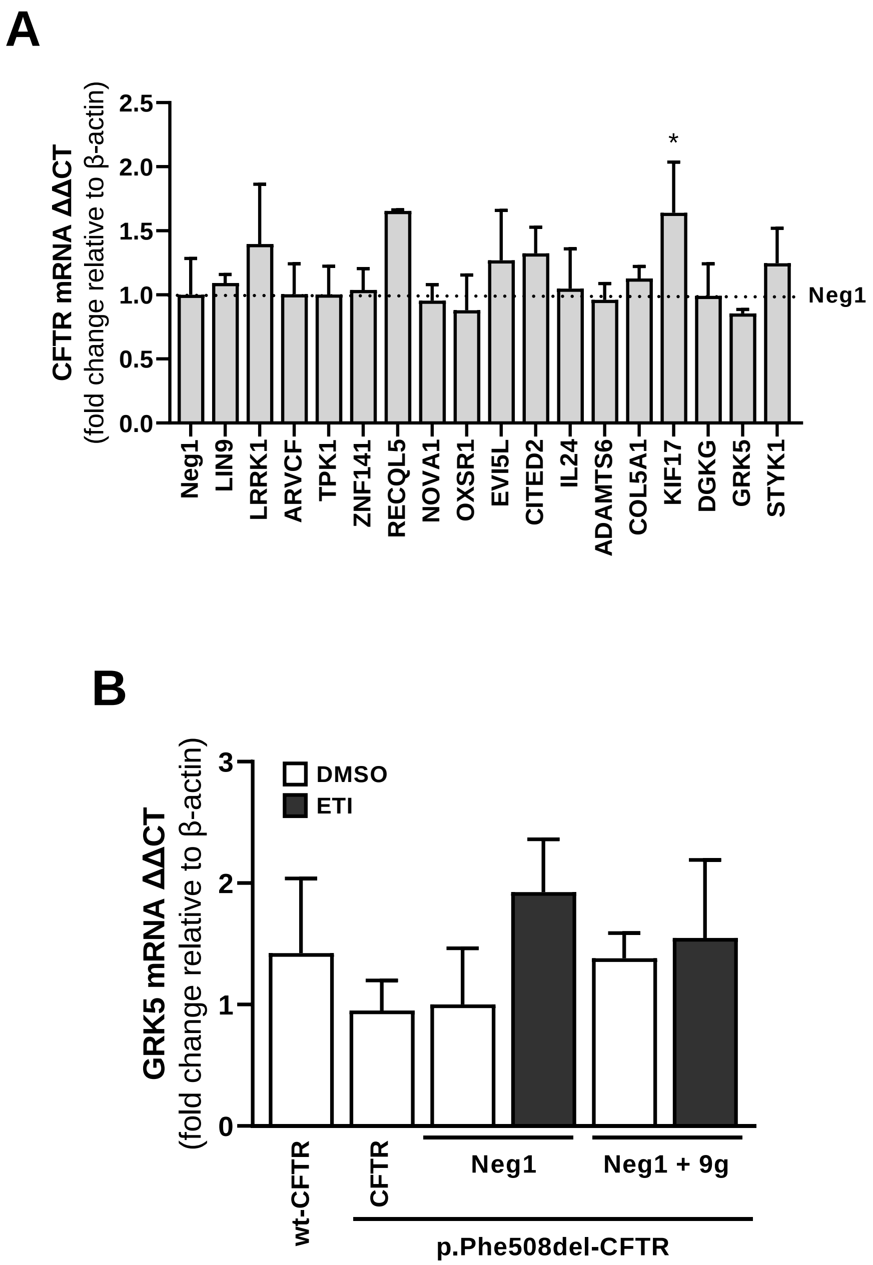

Figure S9. Expression of CFTR and GRK5 mRNA in CFBE cells, related to Figures 3 and 7. (A) RT-PCR quantification of p.Phe508del-CFTR mRNA following knock-down of the 14 best secondary screen hits (LIN9 through KIF17), as well as DGKG, GRK5 and STYK1. This set of siRNAs targets all 5 hit kinases. (LRRK1, TPK1, DGKG, GRK5 and STYK1. Results are ΔΔCT versus the Neg1 negative control, expressed as fold change. Significant CFTR mRNA expression variation was only found when knocking-down KIF17, a borderline p=0.044 in one-way ANOVA followed by Dunnett's post-hoc test (n=3 biological replicates). **(B)** RT-PCR quantification of GRK5 mRNA I cells expressing wt- or p.Phe508del-CFTR following GRK5 inhibition (9g 1µM), in the presence or absence of the VX-770 plus VX-661 plus VX-445 combination (ETI). n = 3-6 biological replicates. No statistically significant differences were observed. All plot values are mean ± SD.

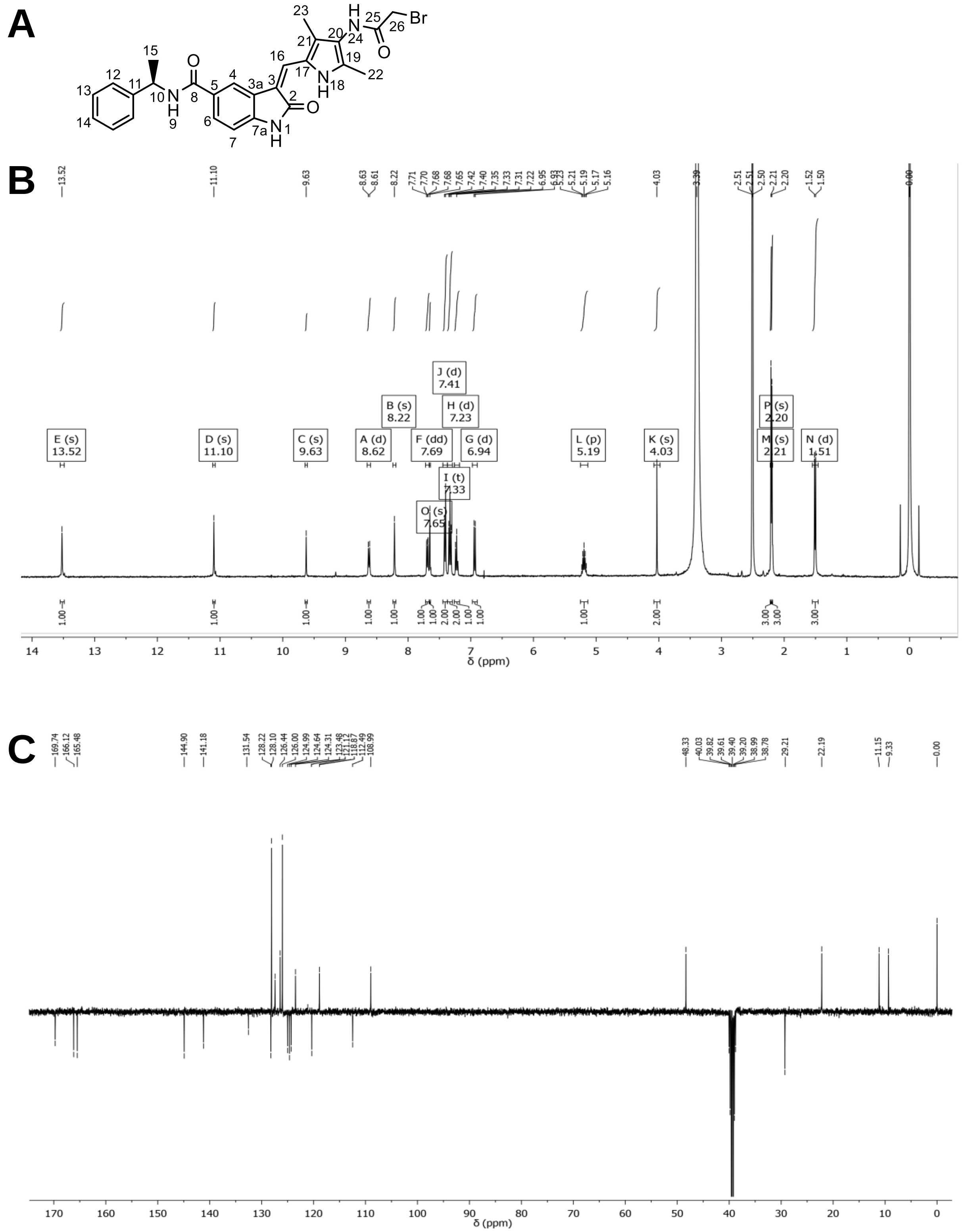

Figure S10. NMR spectra for compound 9g, related to Methods ("9g Synthetic Procedure"). (A) Structure and atom numbering for compound 9g. **(B)** ^{1}H NMR (400 MHz, DMSO-d_6) d 13.52 (s, 1H, H_{18}), 11.10 (s, 1H, H_1), 9.63 (s, 1H,H_{24}), 8.62 (d, J = 7.9 Hz, 1H, H_9), 8.22 (brs, 1H, H_4), 7.69 (dd, J = 8.1, 1.3 Hz, 1H, H_6), 7.65 (s, 1H, H_{16}), 7.41 (d, J = 7.4 Hz, 2H, H_{12}), 7.33 (t, J = 7.6 Hz, 2H, H_{13}), 7.23 (d, J = 7.4 Hz, 1H, H_{14}), 6.94 (d, J = 8.1 Hz, 1H, H_7), 5.19 (p, J = 7.2 Hz, 1H, H_{10}), 4.03 (s, 2H, H_{26}), 2.21 (s, 3H, H_{22}), 2.20 (s, 3H, H_{23}), 1.51 (d, J = 7.1 Hz, 3H, H_{15}). **(C)**^{13}C NMR (101 MHz, DMSO-d_6) d 169.74 (C_2), 166.12 (C_{25}), 165.48 (C_8), 144.90 (C_{11}), 141.18 (C_{7a}), 131.54 (C_{19}), 128.22 (C_{3a}), 128.10 (C_{13}), 127.41 (C_6), 126.44 (C_{14}), 126.00 (C_{12}), 124.99 (C_{17}), 124.64 (C_{21}), 124.31 (C_5), 123.48 (C_{16}),

121.12 (C_3), 118.87 (C_4), 112.49 (C_{20}), 108.99 (C_7), 48.33 (C_{10}), 29.21 (C_{26}),22.19 (C_{15}), 11.15 (C_{22}), 9.33 (C_{23}).

Table S1. Confirmed hit genes and scores (secondary screen), related to Figures 1, 2 and Dataset S3. We defined as confirmed the genes which were hits with 2 siRNAs, considering the primary and secondary screens. The decision took was based on PM CFTR Z-scores. The table presents the scores obtained for every hit siRNA targeting the 35 confirmed genes. The table is ranked by the average PM Z-score of the 2 siRNAs.

		siRNA 1		siRNA 2		
	**Gene**	**ID**	**median PM Z-score**	**ID**	**median PM Z-score**	**Average PM Z-score**
1	LIN9	40573	2.758	s223807	3.675	3.216
2	LRRK1	1680	4.770	s36138	1.345	3.057
3	ARVCF	10246	3.307	s1655	2.135	2.721
4	TPK1	1279	4.007	s25692	1.181	2.594
5	ZNF141	4843	2.588	s15187	2.434	2.511
6	RECQL5	137517	3.269	s17989	1.563	2.416
7	NOVA1	11628	3.127	s9650	1.295	2.211
8	OXSR1	625	3.080	s19304	1.278	2.179
9	EVI5L	37870	2.048	s41855	2.231	2.139
10	CITED2	6450	3.097	s223110	1.098	2.098
11	IL24	5472	2.527	s21673	1.562	2.044
12	ADAMTS6	104129	2.683	s22055	1.300	1.991
13	COL5A1	7983	2.843	s3305	1.096	1.970
14	KIF17	118477	2.195	s33352	1.706	1.951
15	NTNG2	34299	2.597	s39206	1.227	1.912
16	APOB	120962	2.019	s1476	1.714	1.867
17	ZNF384	36530	2.680	s46887	1.009	1.844
18	HELLS	26136	2.655	s6505	1.008	1.832
19	GJB2	46073	2.047	s223724	1.616	1.832
20	VPS26A	15457	2.021	s18360	1.626	1.824
21	LDLRAD3	44082	2.603	s44536	1.043	1.823
22	CLDN4	9704	2.304	s3442	1.333	1.819
23	SLC30A1	117633	2.487	s15331	1.080	1.783
24	ACSBG2	125638	2.112	s37710	1.421	1.767
25	CREBBP	107394	2.267	s3496	1.221	1.744
26	PCDHB2	27448	2.141	s31969	1.275	1.708
27	FOLR2	120092	2.002	s5335	1.400	1.701
28	ISL2	6710	2.161	s35010	1.228	1.695
29	CPZ	104042	2.067	s16226	1.300	1.684
30	DCSTAMP	32873	2.155	s37579	1.142	1.648
31	STYK1	1264	2.216	s30824	1.055	1.636
32	CCL27	18338	2.172	s21303	1.079	1.626
33	QRSL1	119347	2.148	s30619	1.034	1.591
34	GUCY2D	26	2.161	s6383	1.013	1.587
35	CYSLTR2	6093	2.083	s32686	1.051	1.567

Table S2. Effect of top hit genes on p.Phe508del-CFTR processing assessed by WB, related to Figure 3 and S3. Quantification of the WB data obtained when performing KD of confirmed screen hits and selected primary screen siRNAs which did not meet the hit threshold. The table indicates if the gene was part of the confirmed hits, the Ambion Silener Select siRNA catalog number as well as the quantification of p.Phe508del-CFTR band C, B and CFTR processing [C/(B+C)]. ´*´ indicates statistical significance (sig) from cells treated with the non-targeting Neg1 siRNA (p<0.05, one-way ANOVA followed by Dunnett's post-hoc test, n=3). n.a: not available, not performed.

			siRNA 1										siRNA 2									
			ID	Band C			Band B			Processing [C/(B+C)]			ID	Band C			Band B			Processing [C/(B+C)]		
	Gene	In the 35 confirmed hits?		mean	SD	sig	mean	SD	sig	mean	SD	sig		mean	SD	sig	mean	SD	sig	mean	SD	sig
1	LIN9	Yes	s223807	3.39	0.55	*	3.38	0.59	*	1.21	0.28	*	s50204	3.91	0.65	*	3.27	0.58	*	1.47	0.35	
2	LRRK1	Yes	s36138	12.21	2.42	*	3.73	0.10	*	3.80	0.46	*	s36139	18.21	4.60	*	3.51	0.49	*	5.19	0.73	*
3	ARVCF	Yes	s1653	5.00	0.86	*	3.75	0.60	*	1.79	0.44	*	s1655	3.56	0.58	*	3.66	0.58	*	1.36	0.32	
4	TPK1	Yes	s25692	13.78	1.14	*	1.51	0.22		7.63	0.53	*	s25693	11.71	1.81	*	1.27	0.20		5.99	0.46	*
5	ZNF141	Yes	s15186	4.15	0.70	*	3.91	0.67	*	1.44	0.35		s15187	3.68	0.61	*	3.02	0.77	*	1.25	0.29	
6	RECQL5	Yes	s17988	6.46	1.81	*	2.29	0.11	*	2.40	0.28	*	s17989	4.59	1.48	*	1.60	0.11		2.49	0.26	*
7	NOVA1	Yes	s9650	2.74	0.22	*	2.85	0.19	*	1.39	0.21		s9651	2.60	0.20	*	3.10	0.21	*	1.28	0.19	
8	OXSR1	Yes	s19302	2.33	0.18	*	2.89	0.19	*	1.20	0.18		s19304	2.92	0.24	*	2.78	0.18	*	1.36	0.21	
9	EVI5L	Yes	s41855	4.80	0.74	*	4.17	0.33	*	1.20	0.28		s41856	4.42	0.75	*	3.05	0.76	*	1.51	0.36	
10	CITED2	Yes	s2023110	2.05	0.21	*	2.61	0.17	*	1.07	0.09		s20281	1.15	0.11		2.02	0.15	*	0.82	0.06	
11	IL24	Yes	s21673	7.96	1.19	*	2.21	0.10	*	3.58	0.45	*	s21674	2.11	0.46	*	2.11	0.12	*	1.21	0.24	
12	ADAMTS6	Yes	s22054	2.55	0.27	*	9.53	0.91	*	0.42	0.00	*	s22055	0.85	0.06		6.79	0.75	*	0.23	0.01	*
13	COL5A1	Yes	s3305	4.29	0.47	*	1.90	0.24	*	2.10	0.17	*	s3307	2.06	0.19	*	1.39	0.08		1.33	0.09	
14	KIF17	Yes	s33352	7.79	1.18	*	2.29	0.11	*	3.74	0.51	*	s33353	9.72	1.37	*	1.97	0.09	*	3.85	0.51	*
15	NTNG2	Yes	s39206	1.66	0.12		0.73	0.08		1.88	0.28	*	s39208	3.02	0.25	*	2.66	0.17	*	1.61	0.25	*
16	APOB	Yes	s1476	2.62	0.40	*	3.17	0.34	*	1.21	0.28		s1477	3.66	0.60	*	3.83	0.64	*	1.44	0.34	
17	ZNF384	Yes	s46887	5.54	0.78	*	3.10	0.33	*	2.36	0.32	*	s46889	5.28	0.50	*	3.21	0.39	*	1.99	0.14	*
18	HELLS	Yes	s6505	1.92	0.21	*	3.05	0.30	*	0.96	0.07		s6506	1.92	0.26	*	2.30	0.28	*	1.17	0.11	
19	GJB2	Yes	s223724	5.71	1.14	*	2.00	0.12	*	3.95	0.49	*	s5775	8.50	1.12	*	2.00	0.10	*	3.62	0.45	*
20	VPS26A	Yes	s18359	5.68	1.01	*	1.52	0.11	*	3.71	0.47	*	s18360	9.27	2.39	*	1.84	0.10	*	3.87	0.83	*
21	LDLRAD3	Yes	na	na	na	na	na	na	na	na	na	na	na	na	na	na	na	na	na	na	na	na
22	CLDN4	Yes	s3442	5.79	0.68	*	8.77	0.83	*	0.90	0.00		s3443	5.10	0.61	*	9.86	0.94	*	0.88	0.00	
23	SLC30A1	Yes	s15330	2.60	0.28	*	2.08	0.15	*	1.51	0.12		s15331	3.09	0.99	*	2.19	0.32	*	1.91	0.55	*
24	ACSBG2	Yes	s37710	3.34	0.32	*	4.91	0.46	*	0.74	0.00	*	s37711	3.85	0.47	*	5.33	0.70	*	1.15	0.00	
25	CREBBP	Yes	s3495	1.94	0.17	*	2.87	0.16	*	0.97	0.06		s3496	1.66	0.15		1.63	0.18	*	1.11	0.08	
26	PCDHB2	Yes	s31967	2.74	0.22	*	2.81	0.18	*	1.41	0.21		s31969	3.78	0.32	*	2.79	0.18	*	1.73	0.27	*
27	FOLR2	Yes	s5334	3.86	0.48	*	5.02	0.57	*	0.92	0.00		s5335	4.88	0.58	*	7.40	0.70	*	0.94	0.00	
28	ISL2	Yes	s35010	2.57	0.21	*	2.69	0.17	*	1.27	0.19		s35011	3.03	0.29	*	2.90	0.19	*	1.54	0.24	*
29	CPZ	Yes	s16224	5.56	0.55	*	8.91	0.85	*	0.83	0.00		s16226	3.19	0.38	*	7.95	0.75	*	0.53	0.01	*
30	DCSTAMP	Yes	s37577	3.45	0.32	*	1.51	0.20		1.65	0.11	*	s37579	1.15	0.11		1.83	0.03	*	0.98	0.07	
31	STYK1	Yes	s30822	7.40	0.72	*	1.28	0.08		3.49	0.19	*	s30824	8.08	0.79	*	1.69	0.27	*	3.27	0.18	*
32	CCL27	Yes	s21302	2.74	0.13	*	1.41	0.11		1.86	0.07	*	s21303	2.29	0.10	*	1.38	0.05		2.25	0.08	*
33	QRSL1	Yes	s30619	1.34	0.14		1.13	0.21		1.38	0.09		s30620	1.45	0.29		1.29	0.16		1.30	0.20	
34	GUCY2D	Yes	s6381	1.72	0.27		2.03	0.20	*	1.01	0.10		s6383	1.98	0.43	*	2.72	0.26	*	1.02	0.17	
35	CYSLTR2	Yes	s32685	1.49	0.33		1.29	0.10		1.43	0.21		s32686	1.53	0.50		1.31	0.18		1.19	0.32	
36	GRK5	No	s6087	19.91	3.72	*	3.01	0.33	*	5.94	0.90	*	s6089	16.00	4.20	*	3.47	0.85	*	4.51	1.20	*
37	APOA5	No	s42038	18.27	5.52	*	3.21	0.16	*	3.76	1.06	*	s42040	18.92	5.10	*	3.54	0.18	*	3.50	0.55	*
38	KCNIP2	No	s26929	16.09	4.62	*	2.93	0.27	*	5.04	0.93	*	s26931	12.13	2.98	*	3.16	0.38	*	3.37	0.60	*
39	LRP1B	No	s27817	15.91	1.48	*	1.51	0.34		9.23	0.96	*	s28716	13.83	1.06	*	1.36	0.21		7.75	0.56	*
40	NRIP1	No	s15701	9.94	0.74	*	1.31	0.20		6.05	0.46	*	s15703	14.67	1.38	*	1.45	0.26		8.16	0.67	*
41	CCT5	No	s22728	14.10	4.41	*	2.26	0.11	*	3.95	0.93	*	s22729	12.79	4.00	*	2.60	0.02	*	3.13	0.80	*
42	ARL4D	No	s1559	8.64	2.14	*	2.03	0.14	*	3.87	0.68	*	s1560	13.22	2.77	*	3.45	0.39	*	3.03	0.53	*
43	DGKG	No	s3917	11.80	2.17	*	2.29	0.22	*	3.95	0.53	*	s3918	12.83	2.85	*	2.22	0.22	*	4.98	0.68	*
44	FER	No	s5109	11.05	3.37	*	2.33	0.12	*	3.46	0.80	*	s5110	12.52	3.89	*	2.35	0.13	*	3.42	0.80	*
45	GOSR2	No	s18383	9.43	1.95	*	1.29	0.02		7.10	0.40	*	s18385	12.08	1.18	*	1.37	0.08		6.93	0.32	*
46	GJA8	No	s5770	11.48	3.51	*	1.85	0.08	*	4.03	0.96	*	s5771	10.77	3.32	*	2.33	0.13	*	2.97	0.68	*
47	PTGER4	No	s11454	9.28	1.40	*	1.63	0.21	*	4.03	0.54	*	s11455	11.10	2.05	*	2.04	0.12	*	4.16	0.56	*
48	MIER1	No	s33643	4.98	0.97	*	2.01	0.24	*	2.17	0.30	*	s33645	10.72	2.40	*	2.57	0.26	*	3.54	0.62	*
49	EPN3	No	s30053	10.13	2.38	*	2.76	0.30	*	4.52	0.61	*	s30054	10.63	2.10	*	2.76	0.30	*	4.10	0.68	*
50	GDPD5	No	s37599	8.67	1.96	*	2.67	0.28	*	4.15	0.55	*	s37601	9.71	2.15	*	1.88	0.20	*	4.59	0.62	*
51	SCPEP1	No	s34005	8.97	1.35	*	1.88	0.12	*	3.54	0.47	*	na	na	na	na	na	na	na	na	na	na
52	CNR2	No	s3263	8.33	0.91	*	1.22	0.25		5.58	0.61	*	s3265	8.27	0.99	*	1.73	0.04	*	4.65	0.37	*
53	MYH14	No	s36321	7.37	1.80	*	2.00	0.05	*	3.45	0.57	*	s36323	2.66	0.55	*	1.69	0.04	*	1.81	0.24	

Table S3. Specificity analysis of the classification screen, related to Figure 1 and S4 and Dataset S6. Gene KDs which significantly increased PM delivery of the CFTR variants used in the classification screen (hits, Z > +1) which were not hits in p.Phe508del-CFTR. The table is sorted alphabetically, and row numbers are shown.

	wt-CFTR	p.Phe508del-p.Gly550Glu -CFTR	p.Phe508del-p.Arg1070Trp -CFTR	p.Phe508del-4RK-CFTR	DD/AA-CFTR
1	BAG3	ABR	APOBEC3A	ABR	AKT2
2	CACNA1C	ACTR8	ARL4D	APOBEC3A	APOBEC3A
3	CCT8L2	APOBEC3A	CRX	BAG3	ARHGEF2
4	DYRK3	CHRM1	DGUOK	BLK	ATXN1
5	FSD1	CLK1	DNAJB7	CHRM1	BAG3
6	GNG4	CRX	DYRK3	CUL5	CLK1
7	GZF1	DDX5	FGD6	DCP1A	DRD4
8	MMS19	DNAJB7	GNG4	DNAJC8	DRD5
9	MYH14	DRD4	GZF1	GALNS	EMILIN2
10	RELA	EPS15L1	KCNK10	GPR162	ETV4
11	ZNF565	ETV4	LMTK3	KCNK10	FGL1
12	ZNF662	F5	MOB3B	MIER1	GNG4
13	ZNF692	FER	PARD3	MOB3B	GZF1
14		FGD6	TXNDC9	MYH14	LAMA3
15		FHL3		PLA2G12A	LMTK3
16		GOSR2		PLCZ1	MIER1
17		HTR1E		SCPEP1	NAALADL1
18		LAMB2		SLC34A1	NDUFB2
19		LRP1B		SREBF1	PABPC3
20		MAK		ZFP90	PARG
21		MIER1		ZNF19	PDE8A
22		MPST		ZNF692	PHKG1
23		PABPC3			PKMYT1
24		PARG			PRICKLE1
25		PCSK1			PSMA6
26		PHKG1			PTPRJ
27		PMP2			RB1
28		PPP1R1B			SLC25A37
29		PRICKLE1			SLC6A15
30		PTGER4			VWF
31		SEMA6B			ZFP90
32		SGPP1			ZNF678
33		SLC13A1			
34		SLC6A15			
35		SREBF1			
36		TPCN1			
37		TXNDC9			
38		ZFP90			
39		ZNF19			
40		ZNF565			

Table S4. ERQC analysis of the classification screen, related to Figure 1 and S4 and Dataset S6. Gene KDs which significantly increased PM delivery of p.Phe508del-CFTR (Z > +1) but inhibited PM delivery of the indicated variants (Z < -1). The table is sorted alphabetically, and row numbers are shown.

	wt-CFTR	p.Phe508del-p.Gly550Glu-CFTR	p.Phe508del-p.Arg1070Trp -CFTR	p.Phe508del-4RK-CFTR	DD/AA-CFTR
1	ACSBG2	CREBBP	APOB	ACSBG2	CITED2
2	APOB	GJB2	CLDN4	CREBBP	CREBBP
3	CLDN4	LRRK1	COL5A1	DCSTAMP	DCSTAMP
4	COL5A1	PCDHB2	CREBBP	GJB2	LDLRAD3
5	CREBBP	RECQL5	CYSLTR2	GUCY2D	
6	DCSTAMP		DCSTAMP	NTNG2	
7	FOLR2		FOLR2	PCDHB2	
8	GJB2		GJB2	VPS26A	
9	GUCY2D		GUCY2D	ZNF384	
10	IL24		IL24		
11	ISL2		NTNG2		
12	LIN9		PCDHB2		
13	NTNG2		RECQL5		
14	PCDHB2		VPS26A		
15	QRSL1				
16	RECQL5				
17	STYK1				
18	TPK1				
19	VPS26A				

Table S5. Knock-down efficiency of hit kinase genes, related to Figure 4 and 6. CFBE cells were reverse transfected with Ambion Silencer Select siRNAs, either a non-targeting sequence (Neg1) or siRNAs targeting each kinase: si3918 (DGKG), s36138 (LRRK1), s6087 (GRK5), s25692 (TPK1) and s30824 (STYK1). The expression of each gene was quantified through RT-PCR (ΔΔCT versus β-actin) and expressed as the normalized fold change relatively to the corresponding Neg1 treatment. The table shows the mean ± SD quantification, as well as the p-value (two-tailed unpaired Student's *t*-test, considering equal variance across samples). * indicates $p < 0.05$.

	DGKG	GRK5	LRRK1	STYK1	TPK1
**siNeg1**	100.00 ± 21.36 %	100.00 ± 21.75 %	100.00 ± 21.19 %	100.00 ± 41.77 %	100.00 ± 31.20 %
**siGene**	55.38 ± 15.22 %	61.75 ± 0.25 %	40.48 ± 12.84 %	70.51 ± 14.71 %	77.55 ± 4.73 %
**p-value**	0.0421	0.0382	0.0141	0.3130	0.2853
**Significance**	*	*	*		
